# Behavior Change Techniques Within Digital Interventions for the Treatment of Eating Disorders: Systematic Review and Meta-Analysis

**DOI:** 10.2196/57577

**Published:** 2024-08-01

**Authors:** Pamela Carien Thomas, Kristina Curtis, Henry W W Potts, Pippa Bark, Rachel Perowne, Tasmin Rookes, Sarah Rowe

**Affiliations:** 1 Department of Epidemiology & Applied Clinical Research Division of Psychiatry University College London London United Kingdom; 2 Department of Clinical, Educational and Health Psychology University College London London United Kingdom; 3 UCL Institute of Health Informatics University College London London United Kingdom; 4 UCL Cancer Institute University College London London United Kingdom; 5 UCL Research Department of Primary Care and Population Health University College London London United Kingdom

**Keywords:** digital health, eHealth, mobile health, mHealth, mobile apps, smartphone, behavior change, behavior change technique, systematic review, eating disorders, disordered eating, binge eating, bulimia nervosa, mobile phone

## Abstract

**Background:**

Previous systematic reviews of digital eating disorder interventions have demonstrated effectiveness at improving symptoms of eating disorders; however, our understanding of how these interventions work and what contributes to their effectiveness is limited. Understanding the behavior change techniques (BCTs) that are most commonly included within effective interventions may provide valuable information for researchers and developers. Establishing whether these techniques have been informed by theory will identify whether they target those mechanisms of action that have been identified as core to changing eating disorder behaviors. It will also evaluate the importance of a theoretical approach to digital intervention design.

**Objective:**

This study aims to define the BCTs within digital self-management interventions or minimally guided self-help interventions for adults with eating disorders that have been evaluated within randomized controlled trials. It also assessed which of the digital interventions were grounded in theory and the range of modes of delivery included.

**Methods:**

A literature search identified randomized controlled trials of digital intervention for the treatment of adults with eating disorders with minimal therapist support. Each digital intervention was coded for BCTs using the established BCT Taxonomy v1; for the application of theory using an adapted version of the theory coding scheme (TCS); and for modes of delivery using the Mode of Delivery Ontology. A meta-analysis evaluated the evidence that any individual BCT moderated effect size or that other potential factors such as the application of theory or number of modes of delivery had an effect on eating disorder outcomes.

**Results:**

Digital interventions included an average of 14 (SD 2.6; range 9-18) BCTs. *Self-monitoring of behavior* was included in all effective interventions, with *Problem-solving*, *Information about antecedents*, *Feedback on behavior*, *Self-monitoring of outcomes of behavior*, and *Action planning* identified in >75% (13/17) of effective interventions. *Social support* and *Information about health consequences* were more evident in effective interventions at follow-up compared with postintervention measurement. The mean number of modes of delivery was 4 (SD 1.6; range 2-7) out of 12 possible modes, with most interventions (15/17, 88%) being web based. Digital interventions that had a higher score on the TCS had a greater effect size than those with a lower TCS score (subgroup differences: *χ*^2^_1_=9.7; *P*=.002; *I*²=89.7%) within the meta-analysis. No other subgroup analyses had statistically significant results.

**Conclusions:**

There was a high level of consistency in terms of the most common BCTs within effective interventions; however, there was no evidence that any specific BCT contributed to intervention efficacy. The interventions that were more strongly informed by theory demonstrated greater improvements in eating disorder outcomes compared to waitlist or treatment-as-usual controls. These results can be used to inform the development of future digital eating disorder interventions.

**Trial Registration:**

PROSPERO CRD42023410060; https://www.crd.york.ac.uk/prospero/display_record.php?RecordID=410060

## Introduction

### Background

The current eating disorder (ED) treatment model is falling short for patients [1], with a significant majority of people with EDs failing to get help [2]. This may be due to limited access to services [3] and the stigma and shame associated with their condition [4]. EDs have the highest mortality of any psychiatric disorder [5], and they may be long-lasting and may cause physical, emotional, and neurobiological damage if left untreated [6]. The COVID-19 pandemic has further compounded the problem, with a surge in urgent referrals and increased waiting time in an already underresourced system [7]. Action is urgently required to address this treatment gap [8]. A promising strategy that can improve access to evidence-based treatments is the development and implementation of digital interventions. Digital interventions refer to the use of digital technologies, such as mobile apps, websites, or virtual reality, to deliver health care or behavioral interventions.

Advantages of digital interventions include the ability to reach many people at minimal or no additional cost per person, and they can be used at an individual’s convenience, at home, anonymously, and at a self-suited pace [9]. Shame and stigma may make people with EDs more likely to engage in digital interventions to achieve improvements in their symptoms [10,11], and evidence demonstrates that the demand for self-guided digital interventions is growing among people with EDs [11]. While digital self-management interventions are not the only solution to address the existing service gap, they can broaden the dissemination of evidence-based treatments and help more people get support for their condition [12].

Digital interventions for EDs have shown promising evidence in treating ED symptoms [13-15] with results sustained, or even improved, at follow-up [16]. However, our understanding of how these interventions work and what contributes to their effectiveness is limited [17], restricting the potential effectiveness and impact of digital ED interventions. It is widely recognized that digital health interventions should incorporate evidence-based methods and behavior change theory into their development [18]. Theory represents the accumulated knowledge of the mechanisms of action (MOAs; mediators) and moderators of change as well as the a priori assumptions about what human behavior is and what the influences on it are [19]. Using behavior change theory in designing digital health interventions may help pinpoint the factors influencing the target behavior, referred to as *MOAs* in behavioral science. These MOAs, such as knowledge and beliefs, are pathways through which interventions can impact behaviors. Designers can then connect these MOAs to practical elements called “behavior change techniques” (BCTs), which play a crucial role in transforming disordered behaviors into healthier target behaviors. While there are some dissenters regarding such systematization of practice, arguing for the importance of variability, there is general agreement of the value of better descriptions of interventions for clarity and replication [20]. This systematic approach has been applied in the development of effective digital health interventions in areas such as the treatment of addictive disorders, physical activity, and weight loss [21,22], as well as in more clinically oriented interventions, such as diabetes management [23-25]. Specific BCTs have been linked to improved clinical outcomes [26-28] and are a useful means of describing active components within complex digital interventions [29]. The integration of specific BCTs may optimize digital ED treatment interventions, helping achieve significant symptom improvement by addressing those factors (eg, food avoidance, dietary restriction, and body image concerns) that influence common ED behaviors (eg, bingeing and purging).

### Objectives

This review aimed to gain insights from previous randomized controlled trials (RCTs) as to which BCTs may contribute to the effectiveness of digital ED interventions [30]. It focused on RCTs as they have the highest possible level of evidence compared to other study designs and can be used to make causal inferences [31]. It also assessed whether the interventions were grounded in theory, given that theory is a “necessary precursor to the development of effective interventions” [32].

We hypothesized that interventions that specifically targeted the behavioral and psychological aspects of ED via the use of relevant BCTs would be more likely to improve ED outcomes. We also hypothesized that the interventions informed by theory were more likely to be effective. Having multiple modes of delivery (eg, apps, video, and audio) may be associated with enhanced treatment outcomes [15] based on the idea that the diversity offered by multimedia formats might facilitate effectiveness through an enhanced and more engaging user experience [33].

Our specific research questions were as follows:

- Which BCTs are most frequently included in digital interventions for the treatment of EDs that have been evaluated in RCTs? Which BCTs are most frequently associated with effective interventions?
- Are included BCTs informed by theory?
- Which modes of delivery have been adopted to deliver the BCTs?
- Was there evidence to suggest that specific BCTs, or related factors, moderated the intervention effect size?

## Methods

### Search Strategy

The searches were completed across the following databases between April 1, 2023, and June 30, 2023: MEDLINE (Ovid), Embase, PsycINFO, CINAHL, Emcare (Ovid), CENTRAL, Web of Science, and Scopus. The protocol was registered in the PROSPERO database (CRD42023410060). These findings are reported in accordance with the PRISMA (Preferred Reporting Items for Systematic Reviews and Meta-Analyses) guidelines [34]. The search strategy was developed based upon previous similar systematic reviews of digital interventions and EDs [15,35] and in consultation with a specialist librarian at University College London. The search strategy included 2 main concepts based on EDs and types of digital intervention (web based or smartphone). It included a combination of Medical Subject Headings (MeSH) terms and free-text terms. The search was adapted for each database. A Cochrane RCT filter was applied to the search results within relevant databases [36]. Full details of the search strategy can be found in Multimedia Appendix 1.

The first reviewer (PT) initially screened all titles and abstracts for the first phase of the review, and a second reviewer (PB) screened a random 9.98% (375/3758) of the results within Covidence (Veritas Health Innovation). Both reviewers independently screened 100% (79/79) of articles in the final full-text screening stage. Results were compared, and any discrepancy was resolved by discussion. There was a good to excellent degree of interrater agreement (initial screening: κ=0.92 and final screening: κ=0.720).

### Study Selection

Eligible studies were selected by applying the inclusion and exclusion criteria (Textbox 1).

**Inclusion and exclusion criteria.**

**Inclusion criteria**

- Adults in general population
- Self-management interventions and guided self-help interventions for individuals
- Included study participants who meet subthreshold and threshold criteria for an eating disorder
- Stand-alone digital intervention with minimal or some therapist support
- Outcome measure using the Eating Disorder Examination Questionnaire (EDE-Q)
- Randomized controlled trials

**Exclusion criteria**

- Interventions aimed at <16 years old
- Intervention aimed at health care professionals
- Intervention specific to relapse prevention and aftercare
- Intervention specific to eating disorder prevention
- Intervention aimed at obesity and weight management
- Telemedicine or teleconferencing
- Augmentation therapy (app as an *add-on*)
- Digital intervention with intensive levels of supplementary therapist support
- Group cognitive behavioral therapy; group therapy
- Technologies that have been superseded (ie, CD-ROM, vodcast, and SMS text messaging)
- Interventions that used mobile phones but did not involve apps (eg, were based solely on SMS text messaging or emails)
- No clear description of the intervention design (not possible to code for behavior change techniques)
- Qualitative studies
- Feasibility and acceptability studies as well as pilot studies
- No clear outcome measures (using the EDE-Q)

### Data Extraction

The primary researcher (PT) extracted and coded the data for included studies, including author, year, country of origin, study and participant characteristics (number of participants, age, gender, ethnicity, diagnosis, inclusion and exclusion criteria, and dropout rates), and intervention characteristics (intervention description, therapist involvement, BCTs, modes of delivery, duration of treatment, follow-up, and key outcomes). Outcomes data for all the studies were independently extracted by 2 reviewers (PT and TR). Results were compared, and any disagreements were resolved by discussion. Where key data were missing, study authors were contacted for the missing information. A cutoff period of 4 weeks was provided.

### Outcome Measures

The Eating Disorder Examination Questionnaire (EDE-Q) [37] was used as the primary outcome measure of interest, given that it is the National Institute for Health and Care Excellence “gold standard” measure of ED psychopathology and was used as the primary outcome measure in most of the included RCTs. It includes frequency data on key behavioral features of EDs in terms of number of episodes of the behavior (including bingeing and purging), making it a suitable outcome measure for this review [38]. Where reported, changes in the number of objective binge episodes (OBEs) after treatment were examined for consistency, providing complementary data on intervention effectiveness.

### BCT Coding, Modes of Delivery, and Theory Coding Scheme

Each study was assessed for the presence of each of the 93 BCTs using the BCT Taxonomy v1 [30], assessing the number of BCTs in each digital intervention and the frequency of each BCT in the sample overall. The BCT Taxonomy is a hierarchically organized, common language tool for the classification of the *active ingredients* [30] required to bring about change in an intervention. The validity of this approach has been well established, and its reliability and value have been consistently demonstrated across multiple areas since its inception [39-41].

The modes of delivery used within each of the interventions to deliver the BCTs was assessed using relevant components from the Model of Delivery Ontology v2 [42]. If the modes of delivery were changed during the course of the study, the modes of delivery included within the initial study design were coded, as these were appropriate for the outcome measures used.

An adapted version of the theory coding scheme (TCS) [43] was used to evaluate the theoretical basis of the included studies. These adaptations were made in consultation with an experienced behavior change scientist (KC), on the basis that the coding scheme was originally developed for use in a different context and some of the criteria were not relevant. Hobbis and Sutton [44] justified the case for cognitive behavioral therapy (CBT) as an addition to the Theory of Planned Behavior–based interventions; hence, it was considered a valid theoretical basis when used to inform intervention design. All studies were independently coded against these frameworks by 2 reviewers (PT and RC), with any discrepancies resolved by discussion involving a third reviewer (KC). This meant the BCTs were double-blind coded by 2 reviewers across all studies. These results were compared, with a third reviewer involved where necessary to resolve any discrepancies. A briefing document was provided to the second reviewer in advance of coding, which included definitions and examples of BCTs, to ensure reliability. The coding was completed in 2 stages, with the second reviewer coding approximately 30% (5/17) papers first. The coding was compared between the 2 reviewers to identify any inconsistencies in applying the BCT framework, aiming to maximize consistency when reviewing the remaining 70% (12/17) of the papers.

For interventions to be included in the follow-up, they had to be assessed at least 8 weeks after the postintervention period. This time frame allows for a reasonable evaluation of sustained treatment effects and avoids coinciding posttreatment and follow-up evaluations across different studies.

### Data Synthesis

The associations between BCTs and intervention effectiveness were analyzed. A brief narrative synthesis was used to organize and present the data within the text, with a summary of the information extracted from each study, including outcomes reported, BCTs, and other items provided in tabular form.

Frequency counts of the most commonly used BCTs were conducted for both *all interventions* and *effective interventions*, and the results were compared. The effectiveness of an intervention was determined by a statistically significant effect (*P*<.05) on ED behavior change (as measured by the EDE-Q 6.0). In studies with an active comparator, the pre-post outcome data for the intervention arm were examined independently to assess efficacy. These results were then considered in the context of the study design and compared with similar waiting list (WL) control studies. BCTs were considered effective if they were identified in at least 75% (13/17) of effective interventions [18]. A further division of effective interventions was completed based on whether they were effective at postintervention or follow-up.

### Meta-Analytic Procedure

The purpose of this meta-analysis was to pool data across RCT studies regarding the effectiveness of digital interventions compared to waitlist control or treatment-as-usual (TAU) controls at postintervention and follow-up time points to explore what might be contributing to the overall effect sizes, primarily the contribution of any particular BCT. Studies with an active control group, such as face-to-face (F2F) therapy, bibliotherapy, another digital intervention, or day patient programs, as well as studies with missing (EDE-Q total) outcome data were excluded from the meta-analysis.

As a first stage, the meta-analysis procedure calculated pooled estimates of effect sizes (differences in EDE-Q total scores) at postintervention and follow-up time points for waitlist and TAU RCTs and presented these results as forest plots (using RevMan v. 5.4, The Cochrane Collaboration). Effects were based on means, SDs, and sample sizes reported within the studies. The primary outcome was EDE-Q behaviors (dietary restraint, weight concern, shape concern, and eating concern). As the included studies were RCTs, baseline values were not adjusted for across studies, as they would be expected to be similar across treatment and control groups. Due to substantial heterogeneity among the studies, which varied in design (eg, duration of treatment and level of therapist involvement), a random-effects model was used to estimate the weighted pooled effect for each outcome. This approach accounts for the distribution of the true effect across individual studies [45]. The *I*^2^ statistic was used as a measure of heterogeneity, describing the percentage of variation across studies that was due to heterogeneity rather than chance [46]. Heterogeneity >60% was considered substantial [47] and suitable for subgroup analyses. Given that the EDE-Q primary outcome measure was continuous, the mean difference (MD) was used to describe the pooled outcome effects and the overall effect size (*z*-statistic) alongside its *P* value. Sensitivity analysis was completed to check for consistency of the effect size, and publication bias was explored using funnel plots (Multimedia Appendix 2 [13-15,48-56]).

It was then possible to complete subgroup analyses to identify whether there was evidence for any BCTs acting as moderators of effect size. A shortlist of BCTs were identified upfront according to the transdiagnostic theory of EDs by Fairburn et al [57,58]. This was to avoid post hoc analysis of multiple BCTs, which would increase the likelihood of finding significant results through chance. If any of these prespecified BCTs were identified in >75% of effective interventions, they were included in the subgroup analyses: *2.2. Feedback on behavior*, *2.3 Self-monitoring of behavior*, *2.4 Self-monitoring of outcome(s) of behavior*, *4.2 Information about antecedents*, *7.7 Exposure*, and *11.2 Reduce negative emotions*. Additional related concepts were also explored, including mode of delivery (<5 vs ≥5 out of 12 possible sessions), TCS (high vs low), degree of therapist support (none or minimal vs some), and duration of therapy (<8 weeks vs ≥8 weeks). These factors were considered as they could contribute to heterogeneity and impact effect size.

### Risk-of-Bias Assessment

The revised Cochrane Risk-of-Bias tool for randomized trials was used for assessing risk of bias in RCTs with studies assessed against 6 domains [59] (Multimedia Appendix 3 [43]). Risk-of-bias analysis was completed for all articles by PT, with over 20% (4/17) of the articles also being independently assessed by a second reviewer (TR). Disagreements were resolved via discussion. There was a high level of interrater agreement (interrater reliability [IRR]=0.9).

## Results

### Included Studies

**Figure 1:**
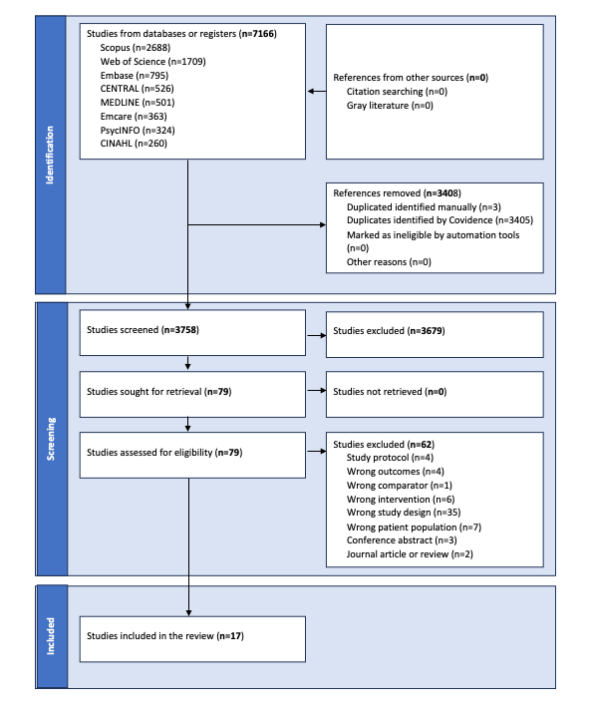
PRISMA (Preferred Reporting Items for Systematic Reviews and Meta-Analyses) flow diagram.

A PRISMA flow diagram (Figure 1) represents the literature search. A total of 17 RCT studies were identified for inclusion in this review.

Of the 17 RCT studies identified, 12 (71%) included a WL comparator (or TAU), with 5 (21%) having *active* controls.

### General Study Characteristics

The 17 studies included 12 (71%) parallel arm trials, 4 (24%) multiple-arm studies [13,48,49,60], and 1 (6%) cluster RCT [50]. Of these, 12 (71%) studies included active treatment compared to a WL control, informational control, or TAU, while 5 (29%) studies compared active treatments to other interventions, including F2F treatment [16], day patient care [61], and other digital treatment interventions [51,62,63].

A total of 9 (53%) studies included all or nearly all female participants (>95%); 5 (29%) studies included 5% to 10% male participants, and 2 (12%) studies included >10% male participants. Ethnicity was not mentioned in 12 (71%) of the 17 studies, with 2 (12%) mentioning nationality but not ethnicity and only 3 (18%) providing any ethnic breakdown. Mean age ranged from 22.1 years [50] to 43.2 years [16] across studies, with participants aged between 17.3 and 55.5 years. The total number of participants overall was 5254, with 1956 included in the meta-analysis (WL and TAU studies only). Inclusion and exclusion criteria were highly variable, with some studies having clear diagnostic criteria that had to be met, excluding participants with comorbidities or with previous experience of inference-based CBT, while others permitted individuals to participate without meeting any diagnostic criteria, provided they were aged >16 years and had access to the internet. One study allowed participants to receive other forms of psychological, medical, or other treatment for their ED, whether in the digital intervention treatment arm or control condition [52].

The studies took place in North America (2/17, 12%) [50,62], Europe (11/17, 65%; Switzerland, Germany, Sweden, Austria, and the Netherlands) [13,15,16,48,49,52-55,64,65], and Australia or New Zealand (4/17, 24%) [14,51,60,63]. The included studies are listed in Multimedia Appendix 4 [13-16,48-55,60-65].

### Summary of Intervention Types and Outcomes

The ED diagnoses included 6 studies focusing on binge eating disorder and binge eating symptoms [15,16,51,52,60,63], 3 studies on bulimia or eating disorders not otherwise specified [13,64,65], and 8 studies concerning individuals with any ED symptoms [14,48-50,53-55,62]. The studies included a number of different interventions (Multimedia Appendix 5 [13-16,48-55,60-65]), with the most common being Salut BED or Salut BN (5/17, 29%) [13,15,16,64,65], Break Binge Eating or Break the Diet Cycle (4/17, 24%) [14,51,60,63], and Featback (2/17, 12%) [48,49].

Studies included internet and mobile-based digital interventions, frequently including messaging or email feedback or prompts. A total of 2 studies focused specifically on an app [14,62], 4 studies included blended internet and smartphone interventions [50,51,60,63], and 11 studies were internet-only interventions. Interventions lasted between 4 weeks and 12 months, with 11 interventions lasting ≤8 weeks and 6 interventions lasting >8 weeks [13,15,48-50,53]. Interventions varied in the number of modules, ranging from 4 to 11, which resulted in differences in the amount of content provided and allowed for varying timescales to complete these modules.

Only studies with digital interventions with no or relatively minimal levels of therapist support (eg, weekly emails) as well as interventions with *some* therapist support were included. This resulted in 4 studies with no therapist involvement [14,48,49,62], 7 studies with *minimal* therapist involvement [13,15,51,52,54,60,63], and 6 studies with *some* therapist involvement [16,50,53,55,61,64].

Outcome measures were most commonly the EDE-Q, although other measures such as the number of OBEs were also frequently reported. Dropout rates at postintervention measurement were between 6.7% and 58% for the digital intervention. They tended to be higher in the interventions with minimal or no support conducted in a community setting, such as those in which participants signed up and participated via an internet service [14,48,49,51,62]. However, design characteristics such as feedback on behavior or feedback on outcomes of behavior also seemed important [51].

The details of the digital interventions within the 17 studies, including their constituent BCTs, are described in Multimedia Appendix 5.

### Study Outcomes at Postintervention and Follow-Up

A total of 11 (92%) of the 12 RCTs that compared a digital intervention to a WL or TAU control demonstrated a significant improvement in ED outcomes (as measured by the EDE-Q) for the digital intervention over the control condition at postintervention, except for the study by Aardoom et al [48]. The WL and TAU control studies that reported the number of binge eating episodes at the postintervention time point (11/12, 92%) [13-15,48,49,51,53-55,60] also reported a significant reduction in OBEs compared to the WL and TAU control condition. All WL and TAU studies that reported follow-up data (9/12, 75%) reported a significant reduction in ED outcomes (EDE-Q total and OBEs) compared to the control condition, including the study by Aardoom et al [48].

When the control condition was an active comparator, of traditional F2F treatment [16] or a day patient program [61], participants in the active comparator arm performed considerably better than the digital intervention at the postintervention time point, but results were comparable at follow-up in both studies. Where the active comparator was a similar digital health intervention, either broader in terms of functionality [51] or consisting of interactive versus static content [62,63], there were no significant differences observed in EDE-Q total outcomes or secondary outcome measures at the postintervention time point (and no follow-up data).

### BCTs in Effective Interventions

A total of 38 (41%) out of 93 BCTs were identified across the clinical content of the interventions (Table 1). The mean number of BCTs per intervention was 14 (SD: 2.57, range 9-18). The following BCTs were reported in >75% (13/17) of effective interventions: *2.3 Self-monitoring of behavior*, *1.2 Problem-solving*, *4.2 Information about antecedents*, *2.2 Feedback on behavior*, *2.4 Self-monitoring of outcomes of behavior*, and *1.4 Action Planning*. *7.7 Exposure* and *11.2 Reduce negative emotions*, which had been predicted to be important, were identified 56% (9/16) and 38% (6/16) of effective interventions, respectively. *5.2 Behavioral practice/rehearsal* (10/16, 63%), *13.2 Framing/Reframing* (10/16, 63%), and *7.1 Prompts/Cues* (9/16, 56%) were present in >50% of effective interventions, suggesting they may also be important in supporting ED behavior change. The IRR was high (IRR=0.84).

BCTs were not identified from the following BCT categories in the taxonomy: *6. Comparison of the behavior*, *14. Schedules consequences*, or *16. Covert learning*. Only 3 studies included a component from the *10. Reward and Threat* category, the *10.4 Social Reward* component.

**Table 1:** Behavior change techniques included in the treatment interventions (by study^a^)

Evaluation of eating disorder studies	[15]	[13]	[16]	[55]	[52]	[62]	[63]	[51]	[60]	[14]	[53]	[49]	[50]	[48]	[54]	[61]	[64]	AI^b^ (n=17), n (%)	EI^c^ (at post intervention; n=16), n (%)
2.3 Self-monitoring of behavior	✓	✓	✓	✓	✓	✓	✓	✓	✓	✓	✓	✓	✓	✓	✓	✓	✓	17 (100)	16 (100)
1.2 Problem solving	✓	✓	✓	✓	✓	✓	✓	✓	✓	✓	✓	✓	✓	✓		✓	✓	16 (94)	15 (94)
4.2 Information about antecedents	✓	✓	✓	✓	✓	✓	✓	✓	✓	✓	✓	✓	✓	✓		✓	✓	16 (94)	15 (94)
2.2 Feedback on behavior	✓	✓	✓	✓	✓			✓	✓	✓	✓	✓	✓	✓	✓		✓	14 (82)	13 (81)
2.4 Self-monitoring of outcomes of behavior	✓				✓	✓	✓	✓	✓	✓	✓	✓	✓	✓	✓	✓	✓	14 (82)	13 (81)
1.4 Action planning	✓	✓			✓	✓	✓	✓	✓	✓	✓		✓			✓	✓	12 (71)	12 (75)
8.1 Behavioral practice or rehearsal	✓		✓			✓	✓	✓		✓			✓		✓	✓	✓	10 (59)	10 (63)
13.2 Framing or reframing	✓	✓				✓	✓	✓	✓	✓			✓		✓	✓		10 (59)	10 (63)
7.1 Prompts or cues				✓		✓	✓	✓	✓	✓	✓	✓		✓				9 (52)	9 (56)
7.7 Exposure	✓	✓	✓	✓			✓		✓				✓		✓		✓	9 (52)	9 (56)
3.2 Social support (practical)	✓		✓	✓	✓							✓		✓	✓		✓	8 (47)	7 (44)
4.1 Instructions on how to perform the behavior				✓	✓		✓	✓	✓	✓			✓	✓				8 (47)	7 (44)
5.1 Information about health consequences					✓		✓		✓		✓	✓		✓	✓	✓		8 (47)	7 (44)
2.7 Feedback on outcomes of behavior									✓		✓	✓	✓	✓	✓		✓	7 (41)	6 (38)
3.1 Social support (unspecified)	✓		✓									✓	✓	✓		✓	✓	7 (41)	6 (38)
8.2 Behavior substitution				✓				✓		✓	✓				✓	✓		6 (35)	6 (38)
11.2 Reduce negative emotions				✓				✓		✓			✓		✓		✓	6 (35)	6 (38)
8.4 Habit reversal				✓		✓		✓		✓			✓					5 (29.4%)	5 (31.3%)
5.6 Information about emotional consequences					✓				✓			✓	✓	✓				5 (29)	4 (25)
8.3 Habit formation				✓			✓						✓			✓		4 (25)	4 (25)
9.3 Comparison of future outcomes	✓		✓			✓			✓									4 (25)	4 (25)
1.1 Goal setting (behavior)				✓	✓	✓												1 (18)	3 (19)
1.3 Goal setting (outcome)				✓	✓	✓												1 (18)	3 (19)
9.2 Pros and cons	✓		✓			✓												1 (18)	3 (19)
12.4 Distraction								✓		✓			✓					1 (18)	3 (19)
15.4 Self-talk	✓	✓											✓					1 (18)	3 (19)
3.3 Social support (emotional)				✓								✓		✓				1 (18)	2 (13)
5.3 Information about social and environmental consequences												✓		✓	✓			1 (18)	2 (13)
10.4 Social reward						✓						✓		✓				1 (18)	2 (13)
5.4 Monitoring of emotional consequences	✓		✓															2 (12)	2 (13)
15.3 Focus on past success				✓		✓												2 (12)	2 (13)
15.1 Verbal persuasion about capability												✓		✓				2 (12)	1 (6)
1.5 Review behavioral goal						✓												1 (6)	1 (6)
1.7 Review outcome (goal)						✓												1 (6)	1 (6)
1.9 Commitment				✓														1 (6)	1 (6)
2.1 Monitoring of behavior by others without feedback											✓							1 (6)	1 (6)
4.4 Behavioral experiments		✓																1 (6)	1 (6)
13.4 Valued self-identity				✓														1 (6)	1 (6)

^a^ Carrard et al [15] (2011), Ruwaard et al [13] (2013), de Zwaan et al [16] (2017), Strandskov et al [55] (2017), Wyssen et al [52] (2021), Tregarthen et al [62] (2019), Linardon et al [63] (2022a), Linardon et al [51] (2022b), Linardon et al [60] (2021b), Linardon et al [14] (2020), Melisse et al [53] (2023), Rohrbach et al [49] (2022), Fitzsimmons-Craft et al [50] (2020), Aardoom et al [48] (2016), Jacobi et al [54] (2012), Högdahl et al [61] (2023), Wagner et al [64] (2013).

^b^ AI: All interventions.

^c^ EI: Effective interventions.

Follow-up data (>8 weeks after postintervention) was available for 9 (53%) out of the 17 studies. In 2 of the studies, there was no data available for the control condition because participants received the intervention. However, since the outcome effects at postintervention were sustained at follow-up, these studies were still included in the analysis [13,52]. A total of 2 studies included an active comparator [16,61], showing improvements on the EDE-Q for the digital intervention arm at postintervention that were sustained or improved at follow-up; hence, they were included in the analysis. This analysis (Table 2) resulted in the following BCTs being identified in effective interventions at follow-up (in >75% of interventions): *2.2 Feedback on behavior, 2.3 Self-monitoring of behavior*, *2.4 Self-monitoring of outcomes of behavior*, *4.2 Information about antecedents*, and *1.2 Problem-solving* (these all were the same at the postintervention time point). The BCTs of *3.2 Social support (practical)*, *3.1 Social support (unspecified)*, and *5.1 Information about health consequences* were more evident in the interventions that were effective at follow-up compared with the postintervention time point. These may be important in sustaining positive outcome effects; however, these findings are based on a small number of studies.

Definitions of the most common BCTs (included in at least 9/17, >50% of interventions), with examples of how they were implemented within the interventions, are included in Multimedia Appendix 6.

**Table 2:** Behavior change techniques included in effective treatment interventions at follow-up (by study).

Evaluation of eating disorder Studies	Carrard et al [15] (2011)	Ruwaard et al [13] (2013)	de Zwaan et al [16] (2017)	Wyssen et al [52] (2021)	Rohrbach et al [49] (2022)	Fitzsimmons-Craft et al [50] (2020)	Aardoom et al [48] (2016)	Jacobi et al [54] (2012)	Högdahl et al [61] (2023)	Effective (at follow-up; 9 studies had follow-up data), n (%)
2.3 Self-monitoring of behavior	✓	✓	✓	✓	✓	✓	✓	✓	✓	9 (100)
1.2 Problem solving	✓	✓	✓	✓	✓	✓	✓		✓	8 (89)
2.2 Feedback on behavior	✓	✓	✓	✓	✓	✓	✓	✓		8 (89)
4.2 Information about antecedents	✓	✓	✓	✓	✓	✓	✓		✓	8 (89)
2.4 Self-monitoring of outcomes of behavior	✓			✓	✓	✓	✓	✓	✓	7 (78)
3.1 Social support (unspecified)	✓		✓		✓	✓	✓		✓	6 (67)
3.2 Social support (practical)	✓		✓	✓	✓		✓	✓		6 (67)
1.4 Action planning	✓	✓		✓		✓			✓	5 (56)
5.1 Information about health consequences				✓	✓		✓	✓	✓	5 (56)
7.7 Exposure	✓	✓	✓			✓		✓		5 (56)
8.1 Behavioral practice or rehearsal	✓		✓			✓		✓	✓	5 (56)
13.2 Framing or reframing	✓	✓				✓		✓	✓	5 (56)

### Theoretical Basis

Nearly all studies (16/17, 94%) reported some level of theoretical basis to their intervention design (Multimedia Appendix 3). Of those that did mention a theoretical basis, CBT and the transdiagnostic theory of EDs were most frequently reported [31,57], sometimes in combination with other theoretical approaches, including acceptance commitment therapy (ACT) and dialectical behavior therapy [51,52]. The description of this theoretical basis was often minimal within the studies; however, these approaches are generally well understood and accepted within ED treatment, and further literature was often referenced [57] to support their use.

Of the 17 studies, 13 (77%) mentioned a target construct as a predictor of behavior (eg, emotional regulation and body image concerns) and designed interventions that targeted these constructs to change ED behaviors. A total of 13 (77%) of the 17 studies reported how theory or predictors were used to select or develop BCTs. However, this was not often done explicitly; instead, interventions typically listed features alongside their theoretical constructs (eg, emotional regulation—access to an emotions tracker and body image concerns—an exercise to break avoidance patterns). Only 4 studies used theory or predictors to tailor interventions to participants [53,55,62,63] based on their specific eating-related concerns.

### Modes of Delivery

The mean number of modes of delivery per intervention was 4 (SD: 1.6, range 2-7) out of 12 possible modes (Multimedia Appendix 3). All interventions included textual information, after which the most common mode of delivery was website (15/17, 88% of studies). Mobile apps were included in just 6 (35.3%) of the 17 studies. Of the 6 studies, 4 (24%) included both website and app modes of delivery [50,51,60,63] and 2 (12%) were app only [14,62].

Video and audio modes of delivery were identified in only 18% (3/17) and 29% (5/17) of the apps, respectively, suggesting rather limited use of multimedia functionality within the interventions, with a greater reliance upon textual information. In 47% (8/17) of the studies, an at-a-distance mode of delivery involving human interaction was included. This typically involved therapists providing weekly feedback on behaviors and assignments delivered via SMS text messaging (10/17, 59%) or email (9/17, 53%). Email was also used to *check-in* with participants to ensure engagement with the intervention. Although phone was used in 24% (4/17) of the interventions, this was usually only if the user was not engaging in the service at risk of dropout, rather than being part of the service.

It should be noted that Fitzsimmons-Craft et al [50] changed their study design after 1 year, based on performance in the first year, from a web-based intervention to an app-based intervention. Given that the outcomes at 1 year were used in the analysis, the app mode of delivery was not coded.

### Risk of Bias

Most studies (15/17, 88%) reported an adequate method of randomization, frequently including computer-generated randomization sequence, although assessors were not always blind to treatment allocation. Most studies (16/17, 94%) reported adequate blinding of outcome assessment, either through the use of web-based self-report outcome assessments or through F2F or phone assessments, in which assessors were blind to treatment allocation. No studies reported blinding participants to the digital intervention, which would have been difficult to achieve. However, none discussed how this lack of blinding might have biased the self-reported outcomes.

The domain where studies scored lowest was in terms of missing outcome data (13/17, 77% studies), which was due to the relatively high attrition rates across studies. Some studies deviated from their analysis plan, including alternative statistical methods in their analysis [49]. Although these may have been justified, they introduced *some concerns* in how those studies had been analyzed and the data that were reported. There was also selective reporting of the results in 5 (29%) of the 17 studies, which put them at a higher risk of bias (Multimedia Appendix 3). When a subgroup analysis was conducted between studies with low or some concerns regarding bias and those at high risk of bias, no significant differences in outcomes were observed (Multimedia Appendix 2).

### Results From Meta-Analysis

**Figure 2:**
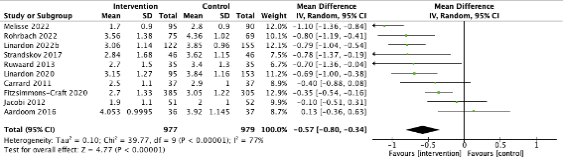
Forest plot showing the mean difference in outcomes (Eating Disorder Examination Questionnaire) for digital eating disorder interventions versus waiting list and TAU controls (at the postintervention time point). TAU: treatment as usual.

We used the MD of EDE-Q total scores as the primary estimate of effect size for each intervention. A total of 10 studies were included with WL or TAU control with EDE-Q outcome data at the postintervention time point. Although 12 studies had a WL or TAU control, one study was excluded due to missing outcome data on the dietary restraint subscale [60] and another was excluded as the control group was given the intervention at 4 weeks; hence, the study comparison at the postintervention time point was against 8 weeks versus 4 weeks active treatment [52]. The pooled effect sizes for the comparison between digital ED interventions and WL or TAU control groups was moderate and statistically significant in favor of the treatment group for ED psychopathology (MD=–0.57, 95% CI –0.080 to –0.39; *Z*=4.77; *P*<.001). Heterogeneity was high (*I*^2^=77%), making it sensible to conduct subgroup analyses (Figure 2).

Sensitivity analysis was completed by removing studies one at a time to consider the impact on effect size, but this did not change the results significantly. There was no clear evidence of publication bias based on a relatively even distribution of studies around the summary estimate line (Multimedia Appendix 2).

It should be noted that while data used for this meta-analysis did not demonstrate statistical significance for the studies by Jacobi et al [54] or Carrard et al [15], when baseline values were adjusted for (as in the original papers), outcomes significantly favored the interventions compared to the control at the postintervention time point in both studies (*P*<.001). Therefore, the interventions were considered to be effective at the postintervention time point. Baseline values were not adjusted for within the meta-analysis based on the assumption that randomized controlled studies should not have baselines differences.

### Moderator and Subgroup Analyses

A total of 6 moderator analyses were conducted to investigate differences in EDE-Q total pooled effect size according to the presence or absence of BCTs in digital interventions. None of the subgroup analyses of BCTs explained any of the heterogeneity of effect sizes across the studies, suggesting that there were other factors that explained this heterogeneity (refer to the example in Multimedia Appendix 1). Heterogeneity within BCT subgroups was also moderate, confirming that there were likely to be other factors explaining this variability.

**Figure 3:**
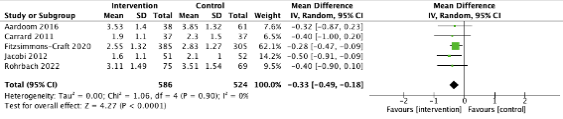
Forest plot showing the results of meta-analysis from theory coding scheme subgroup analysis. TAU: treatment as usual.

Digital ED interventions that had a higher score on the TCS had a greater effect size than those with a lower TCS score (Figure 3). Subgroup analyses showed that interventions that were more highly grounded in theory (high TCS mean=–0.86, 95% CI –1.06 to –0.66; *I*^2^=37%) were significantly more effective than those that had a low theoretical basis (low TCS mean=–0.36, 95% CI –0.61 to –0.11; *I*^2^=56%; subgroup differences: χ^2^_1_=9.7; *P*=.002; *I*²=89.7%; Multimedia Appendix 2). This was the only statistically significant moderation effect that emerged from the subgroup analyses.

There were no significant differences across other subgroup analyses. All subgroup analyses are presented in Multimedia Appendix 2.

### Follow-Up

**Figure 4:**
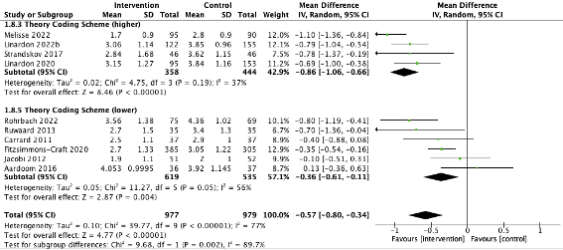
Forest plot showing the mean difference in outcomes (Eating Disorder Examination Questionnaire) for digital eating disorder interventions versus waiting list and TAU controls at follow-up. TAU: treatment as usual.

Only 5 studies with WL and TAU control included EDE-Q outcome data at follow-up (>8 weeks) [15,48,50,54]. The results were significant, with reduction in ED psychopathology favoring the treatment arm (MD=–0.33, 95% CI –0.049 to –0.18) and an overall effect size of *z*=4.27 (*P*<.001). There was no heterogeneity (*I*^2^=0%; Figure 4).

A total of 2 studies were considered high risk of bias, and the remaining 3 studies had *some concerns* due to missing data and selective reporting of the study data; hence, these data should be interpreted cautiously. Given the limited number of studies with outcome data at follow-up (and lack of heterogeneity), subgroup analyses were not completed.

## Discussion

### Principal Findings

There is good evidence to support the efficacy of digital interventions (mainly websites) for people with mild to moderate EDs, with 16 (94%) out of the 17 studies demonstrating efficacy at the postintervention time point, strengthening findings from previous reviews [15,35,56,66]. Effects appear to be maintained at follow-up, with some studies demonstrating continuous reduction in bingeing and purging symptoms with effect sizes similar to those observed in F2F treatment [16,61]. There were few studies on smartphone-based interventions (*apps*); hence, data on their effectiveness as self-management tools, or as guided interventions, remain limited, and further research is required.

Interventions included an average 14 (SD 2.6; range 9-18) BCTs, which compares favorably with other reviews of digital behavior change interventions [67,68], demonstrating that existing interventions already incorporate BCTs to help change ED behaviors. Across the various interventions reviewed, there was a high level of agreement regarding BCTs that were included, which were *Self-monitoring of behavior*, *Self-monitoring of outcomes of behavior*, *Information about antecedents*, *Problem-solving skills*, and *Feedback on behavior*. These are in line with the principles of CBT-ED and the transdiagnostic theory of EDs by Fairburn [57]. Other CBT-ED–related BCTs of *Exposure*, *Cognitive restructuring*, and *Reducing negative emotions* also ranked moderately highly, although there may be an opportunity to integrate these techniques further within digital interventions based on their relevance in treating patients with EDs. While *Prompts or cues* were present in just 56% (9/16) of effective interventions, these techniques may be important to facilitate user engagement within digital interventions [69], which is important if these interventions are to be effective for a greater number of people by reducing dropout rates.

Some effective interventions (3/16, 19%) included additional techniques that are often used in therapy, such as *Distraction* and *Pros and cons*; however, there was insufficient evidence to evaluate if these helped contribute to intervention effects. There were no BCTs in the categories of *Comparison of the behavior*, *Scheduled consequences*, or *Covert learning* across the digital ED interventions, and *Reward or Threat* techniques were rarely used. There is an opportunity to explore how these could be used, potentially learning from other areas of digital health behavior change and testing some of these techniques with potential users. At follow-up, it seemed that social support may be important in supporting a sustained outcome effect [15,48,50], achieved through personalized feedback, encouragement, and practical advice provided within the intervention. This enabled users to achieve greater self-awareness, improved coping skills, greater accountability, and the development of a more supportive social network to assist them in their recovery.

There was no indication that individual BCTs were responsible for differences in outcome effects. This may have been due to the limited number of studies, the small numbers of participants, other factors accounting for the heterogeneity, and the similarity of digital intervention characteristics. It is also because of the study designs, which did not facilitate direct comparison of intervention components across studies. A different approach to design involves using a factorial RCT guided by the Multiphase Optimization Strategy [70], which enables the simultaneous evaluation of multiple variables (eg, BCTs and modes of delivery) and their interactions, without the need for a large sample size. Most studies used CBT-based internet interventions (and some used the same or similar interventions, eg, Salut BN or Salut BED and Break Binge Eating); hence, it could be that there was insufficient variability in the BCTs across studies, making it difficult to detect an association between the most commonly reported BCTs and treatment outcomes. It is also most likely that a combination of BCT inclusion, dose, mode of delivery, and theoretical basis may be important for intervention effectiveness alongside other key design characteristics. Further studies are required to better understand how these factors interact to achieve their effects.

Nearly all studies (16/17, 94%) referred to a theoretical basis for their intervention design; however, they differed to the extent to which theory had been rigorously applied. Most interventions (16/17, 94%) were based on CBT, informed by the transdiagnostic theory of EDs by Fairburn [57], although some interventions also incorporated techniques from ACT and dialectical behavior therapy [51,55]. Interventions that were informed by theory seemed to have a greater effect size within the meta-analysis, consistent with what was hypothesized. They were designed to target those specific MOAs (eg, dietary restraint, body image concerns, and emotional dysregulation) that have been identified as important in changing ED behaviors. However, this result should be interpreted with caution due to the small number of studies, other factors that could explain this result, and the remaining heterogeneity within these subgroups requiring further explanation.

The mean number of modes of delivery per intervention was 4 (SD 1.6, range 2-7) out of 12 possible modes, with a heavy reliance upon textual information and a limited use of audio and video to deliver the BCTs. Nearly half of the interventions (8/17, 47%) included some degree of human interaction, delivered *at a distance*; however, there was no evidence that therapist involvement moderated effect size. There was also no evidence that an increased number of modes of delivery moderated any outcome effect. A key finding was that 2 (12%) of the 17 interventions were app only, suggesting that we require more evidence on app-only approaches with no or minimal therapist support. At the time of this review, the technology used across interventions was relatively homogeneous; hence, we focused on modes of delivery to capture differences in how the interventions were delivered. As technology evolves, it may be important to consider the type of technology used, such as artificial intelligence, as an additional moderating factor.

### Comparison With Prior Work

These results strengthen findings from previous meta-analyses, which provide initial evidence for the effectiveness of digital interventions for reducing ED symptoms [15,35,71,72]. Loucas et al [66] found small effects in their review of internet-based treatments for EDs (n=20), but with the inclusion of more recent studies, small to moderate effects have consistently been demonstrated, with some participants showing significant improvement in ED behaviors at postintervention [53] and at follow-up [15].

Results are consistent with a previous systematic review of mobile health (mHealth) interventions for EDs [73] that concluded that mHealth interventions, either as a self-management tool or complementary to F2F therapy, had limited support. Previous qualitative research has highlighted the promise of such interventions, with high levels of interest in mobile apps and level of acceptability [11,74], although the number of RCTs that demonstrate efficacy remains limited [14,62]. Specific advantages have been identified by patients and clinicians, such as better supporting the real-time logging (food and mood), tracking and feedback to users, reminders to increase adherence to the intervention [17], and the opportunity for just-in-time interventions when an individual may be an elevated risk of engaging in an *unhealthy* behavior (eg, purging) [75]. Research to translate these ideas into effective ED apps that have a place in treatment is still ongoing.

Although this study found that increased levels of multimedia within the digital interventions did not mediate intervention effects, previous research [15] did find that studies with increased use of multimedia channels (audio, video, etc) were associated with greater improvement in ED symptoms. Barakat et al [76] performed a more robust analysis of multimedia channels, analyzing data from surveys returned by the study authors and incorporating additional components such as quizzes and homework, which provided a more detailed and accurate reflection of multimedia inclusion, despite including a range of study designs. Their findings need to be replicated by including more recent RCTs, especially given that they included older studies, some of which were based on now-obsolete technologies (ie, CD-ROM and vodcasts). Interactivity alone is unlikely to meaningfully affect key outcomes in internet-based interventions; instead, it will likely be a combination of interactivity and other design characteristics, such as the quality of intervention content, personalization, persuasive design, or therapeutic alliance principles [7], which are important determinants of outcomes. It could also be that certain populations, such as those with neurodiversity (eg, autism spectrum disorder [77]), benefit more from increased levels of multimedia within digital interventions.

### Strengths

We only included studies using an RCT design, which has not been the case in previous reviews [15,35]. This is the first study to systematically review the BCTs within digital ED interventions, providing greater insights and a more comprehensive picture to inform intervention design and evaluation. Studies that included blended interventions or high levels of therapist support were excluded to allow a thorough analysis of the BCTs within digital interventions and how these may be specifically contributing toward symptom improvement. This study evaluated the effect of BCTs, modes of delivery, and theoretical underpinning on intervention outcomes quantitatively as well as narratively to enable a rigorous evaluation of the data. A large number of databases were searched to ensure that all relevant studies are included in this review, and we found 9 studies that were not included in previous similar reviews. This study has furthered our understanding of how to develop effective digital interventions, providing an opportunity to develop new or improved mHealth interventions for EDs that have the potential to be effective.

Most participants in these studies were recruited from within a community setting; hence, they should be reflective of those with ED in the population who may not currently be getting help from clinical services. This is especially important given the significant increase in demand for ED services since the pandemic [7] and a sustained move to the use of more digital services.

### Limitations

Only 10 studies were suitable for inclusion in the meta-analysis, restricting the power required to detect significant moderating effects of BCTs. Some studies included a small number of participants; hence, it might be underpowered to demonstrate significant differences compared to control groups or significantly affect the meta-analytic findings due to low weight. CIs in several of the studies were relatively large, limiting the ability to find significant results across the pooled studies.

Given that the meta-analysis only examined differences in effect sizes between the digital interventions and control at postintervention and follow-up without including baseline values, it did not assess whether the observed differences were clinically meaningful. In addition, use of the EDE-Q may not have provided a clear picture of all changes in ED behaviors, as not all compensatory behaviors are adequately covered by the EDE-Q [78]. For example, it is possible that participants replaced purging with nonpurging compensatory behavior, such as excessive physical exercise, dieting, and fasting. Studies using self-reported measures of outcomes may not have accurately reflected actual outcomes being subject to self-reporting bias.

Dropout rates in some of the studies was high, varying from 6.7% to 58%. Although studies typically assessed differences in baseline characteristics between those who completed and those who dropped out and typically found minor or no differences, the proportion of participants across who did not complete treatment and provide postintervention assessments is a significant limitation.

We did not have access to the interventions; hence, the BCT coding was based on descriptions of interventions that were available in the public domain (ie, journal publications, supporting information, etc) and some discussion with authors. Studies often did not go into much detail about the theoretical basis upon which the interventions were developed; hence, we were limited in terms of the information that could be coded. While there was a high level of IRR (IRR=0.83), there was an element of subjectivity in how the BCT Taxonomy was interpreted and applied. The theoretical coding scheme used was abbreviated for the purposes of this review and has not been externally validated. There was limited follow-up data; hence, it was not possible to evaluate the effectiveness of the interventions over the longer term (nor complete subgroup analyses). Some BCTs may have helped specific user populations, but studies did not report on outcomes for specific populations, limiting our understanding of what worked for whom. While it is helpful to categorize interventions based on their BCTs, the BCT Taxonomy v1 may be inadequate for identifying all active ingredients that might be contributing to the effectiveness of an intervention, such as those included within ACT.

### Further Work

Further research is needed to evaluate the effects of specific BCTs and combinations of BCTs to identify which are most crucial for improving outcomes in digital interventions for EDs. This research should also explore who benefits most from these techniques and which modes of delivery are most effective. A factorial experiment would allow different combinations of BCTs to be tested to see which combinations, as well as the effect of different modes of delivery, are the most effective [70]. Greater consistency in RCT design would be helpful to maximize the learnings that can be gained as to what is effective, as there continues to be considerable heterogeneity across study designs. Existing digital programs for EDs typically involve numerous strategies, techniques, or modules designed to target a range of behavior change mechanisms, such as restrictive eating, mood dysregulation, body image concerns, and low self-esteem deficits [16,50]. Therefore, further research is required into how to tailor interventions to better meet the needs of individual patients or user *clusters* [79]. Receiving intervention content that is not relevant to a user’s symptom profile may lead to issues with motivation, engagement, and dropout [80]. One way in which this could be explored is via bandit trials, which are a type of adaptive intervention design that allow for personalized treatment allocation based on individual responses. Treatment outcomes across the different intervention options could be evaluated, with a further analysis to determine which treatment options are most effective for which individuals.

The BCT Taxonomy v1 has since evolved into an ontology [81], which could be applied to help identify any additional techniques, such as in ACT, which may not have been accounted for in this review. We did not analyze the dose of BCTs by coding the frequency of each BCT within interventions. This decision was made to avoid adding an additional layer of complexity to this review. Further research could explore whether there is an optimal dose for BCTs.

There is some evidence to suggest that some specific BCTs may improve the user experience and adherence to treatment, which could be explored via further qualitative research, including the way in which BCTs are translated in an intervention. It also may be worthwhile to get user feedback on those BCTs that have rarely been incorporated into digital ED interventions to establish if they may be beneficial to users. This should include those that are commonly used within therapy but are not widely implemented within digital interventions. Further work is required to understand how to leverage the benefits of mobile apps, such as enabling real-time data capture and the opportunity for just-in-time intervention [75] at the point of need.

These studies included minimal or no therapist support. The study by Aardoom et al [48] suggests that self-guided interventions can be effective with automated feedback, while some therapist involvement improves user satisfaction. In depression and anxiety, studies show that treatment programs with some level of guidance are more effective compared to those without some level of guidance [82,83]. More work is required to understand what level of support is optimal, how it benefits users, and the cost-effectiveness of additional support [84]. Research into what level and type of therapist interactions are sufficient to develop any therapeutic alliance within digital ED interventions requires further study, given that therapeutic alliance has been shown to be positively associated with treatment outcome in both F2F treatment [85] and internet-based treatment [86]. This includes research into the use of artificial intelligence chatbots and how they might support the establishment of an alliance [87].

There remains a lack of studies of digital ED interventions involving older people, men, and those belonging to sexual and ethnic minority groups [17]. There is evidence to suggest that ethnicities may have differing requirements from an ED intervention, and these populations may also be less likely to access treatment [72]. It is important that these groups are represented in future research on digital health interventions in EDs from the outset [35] to support the design and development of more accessible and inclusive digital interventions.

Further research is required to understand exactly where these interventions should fit in the treatment pathway to complement the work of ED therapists and health care professionals in this field. It is crucial that this research is translated into *real-world* interventions to offer more evidence-based apps to people with mild to moderate EDs [88,89]. However, it is important to recognize that these apps may not be suitable for everyone and that health care professional support may still be necessary at some stage.

### Conclusions

There is increasing evidence for the effectiveness of digital interventions for the treatment of people with mild to moderate EDs, with improved outcomes at postintervention and sustained outcomes at follow-up time points. Effective digital ED interventions mostly used the same specific BCTs, informed by theory; however, there was no evidence that any 1 BCT contributed to improvements in ED behaviors. However, the presence of self-monitoring in 100% of effective interventions suggests that it may be important for enabling ED behavior change. There seems to be an opportunity for further refinement of BCTs within digital interventions to improve intervention effectiveness by applying learnings from what works in therapy and conducting factorial experiments.

The interventions that were informed by theory and where theory had been applied to identify mechanisms of change and select specific BCTs within the intervention had better outcomes. There was no evidence that increasing the number of modes of delivery had an impact on effect size. There were few studies that evaluated digital apps, indicating potential for the development of higher-quality, evidence-based apps to enhance access to treatment. Future interventions should be grounded in theory targeting those specific mechanisms of change which are important for improving individuals’ ED behaviors.

## Appendix

Multimedia Appendix 1 Search strategy.

Multimedia Appendix 2 Supplementary subgroup analyses from the meta-analysis.

Multimedia Appendix 3 Results of the theory coding scheme, modes of delivery, and risk-of-bias analyses.

Multimedia Appendix 4 Summary of the study characteristics.

Multimedia Appendix 5 Summary of the interventions including behavior change techniques and modes of delivery.

Multimedia Appendix 6 Most common behavior change techniques, definitions, and implementation examples.

Multimedia Appendix 7 PRISMA checklist.

## Abbreviations

ACT: acceptance commitment therapy
BCT: behavior change technique
CBT: cognitive behavioral therapy
ED: eating disorder
EDE-Q: Eating Disorder Examination Questionnaire
F2F: face-to-face
IRR: interrater reliability
MD: mean difference
MeSH: Medical Subject Headings
mHealth: mobile health
MOA: mechanism of action
OBE: objective binge episode
PRISMA: Preferred Reporting Items for Systematic Reviews and Meta-Analyses
RCT: randomized controlled trial
TAU: treatment as usual
TCS: theory coding scheme
WL: waiting list
