# Supplementary material for: Behavior Change Techniques Within Digital Interventions for the Treatment of Eating Disorders: Systematic Review and Meta-Analysis
Source: JMIR Ment Health. 2024 Aug 1;11:e57577. doi: 10.2196/57577 (PMC11327638; doi:10.2196/57577)
Supplement: Multimedia Appendix 1 [file mental_v11i1e57577_app1.docx]

**Table S1.** Key Search Concepts

| Concept 1: | Eating disorder OR Anorex* OR Bulim* OR Binge Eating Disorder OR Binge Eating OR EDNOS OR OSFED OR Disordered eating |
| --- | --- |
| Concept 2: | Internet intervention OR Web-based intervention OR Online treatment OR Online intervention OR Computer assisted therapy OR eTherapy OR smartphone app OR mobile app OR mHealth OR eHealth OR cell phone OR cellular phone. |

(Example from Medline)

1 randomized controlled trial.pt.

2 controlled clinical trial.pt.

3 randomized.ab.

4 placebo.ab.

5 drug therapy.fs.

6 randomly.ab.

7 trial.ab.

8 groups.ab.

9 1 or 2 or 3 or 4 or 5 or 6 or 7 or 8

10 exp animals/ not humans.sh.

11 9 not 10

12 (eating disorder* or anorex* or bulim* or binge eating* or EDNOS or OFSED or disordered eating).mp. [mp=title, book title, abstract, original title, name of substance word, subject heading word, floating sub-heading word, keyword heading word, organism supplementary concept word, protocol supplementary concept word, rare disease supplementary concept word, unique identifier, synonyms, population supplementary concept word, anatomy supplementary concept word]

13 ((internet adj1 (based or intervention* or tool* or treatment* or therapy)) or (web adj1 (based or intervention* or tool* or treatment* or therapy)) or (online adj1 (based or intervention* or tool* or treatment* or therapy)) or computer assisted therapy or computer-based therapy or eTherapy or mobile or mHealth or eHealth or cellphone or cellular phone or digital or app or apps or internet).mp. [mp=title, book title, abstract, original title, name of substance word, subject heading word, floating sub-heading word, keyword heading word, organism supplementary concept word, protocol supplementary concept word, rare disease supplementary concept word, unique identifier, synonyms, population supplementary concept word, anatomy supplementary concept word]

14 exp "Feeding and Eating Disorders"/

15 Internet-Based Intervention/

16 *Telemedicine/

17 Mobile Applications/

18 Internet/ 81092

19 12 or 14 72005

20 13 or 15 or 16 or 17 or 18

21 19 and 20 and 11
