# Supplementary material for: Behavior Change Techniques Within Digital Interventions for the Treatment of Eating Disorders: Systematic Review and Meta-Analysis
Source: JMIR Ment Health. 2024 Aug 1;11:e57577. doi: 10.2196/57577 (PMC11327638; doi:10.2196/57577)

**Figure S1.** BCT Subgroup Analysis Example: Includes / Excludes “13.2 Framing / Re-framing”


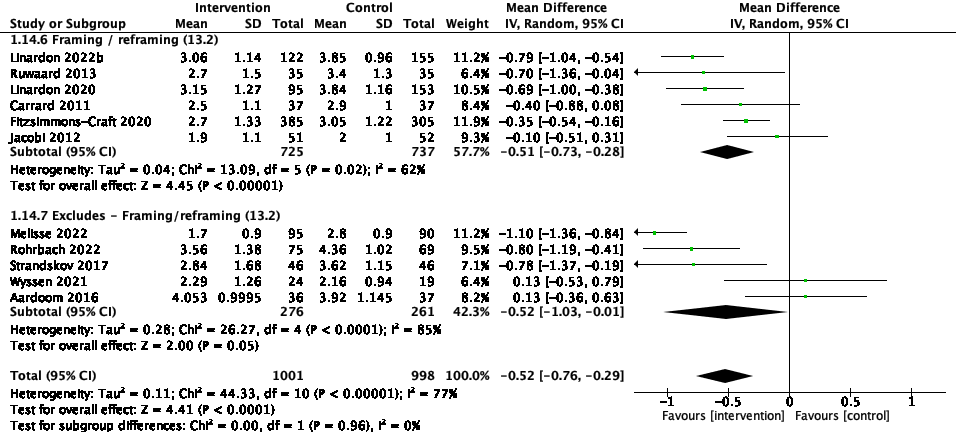


**Figure S2.** Modes of Delivery Sub-Group Analysis

**
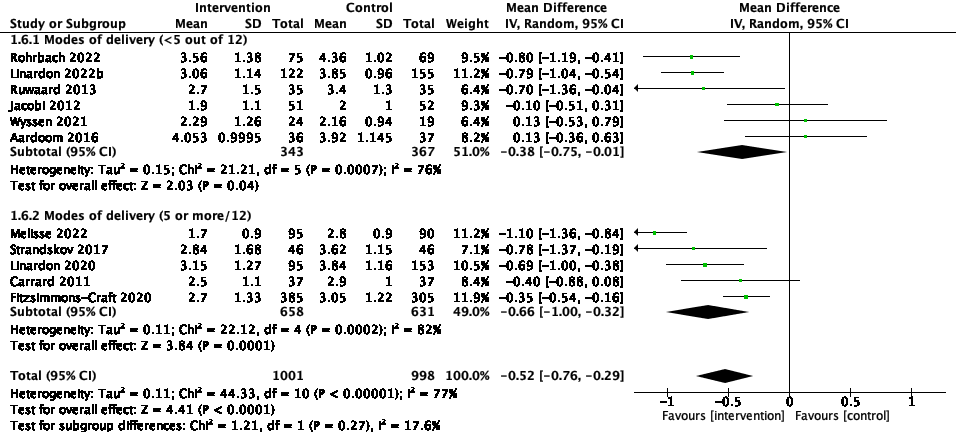
**

**Figure S3.** No/Low Therapist Involvement vs. Some Therapist Involvement Sub-group Analysis


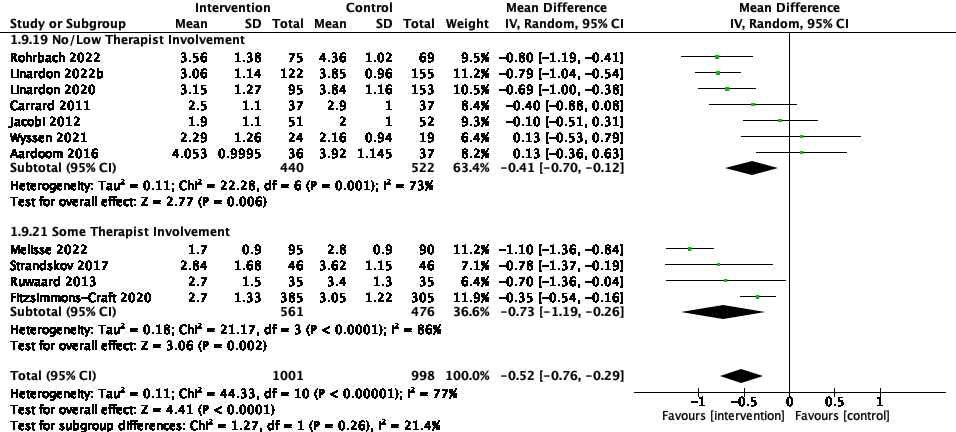


**Figure S4.** Duration of Therapy (8 weeks or less; >8 weeks)


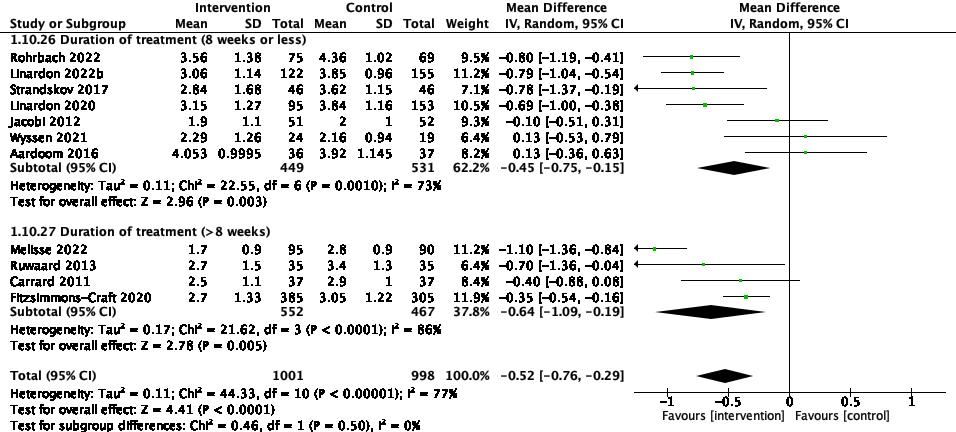


**Figure S5.** Risk of Bias (Low Risk/Some Concerns; High Risk) Subgroup Analysis

**
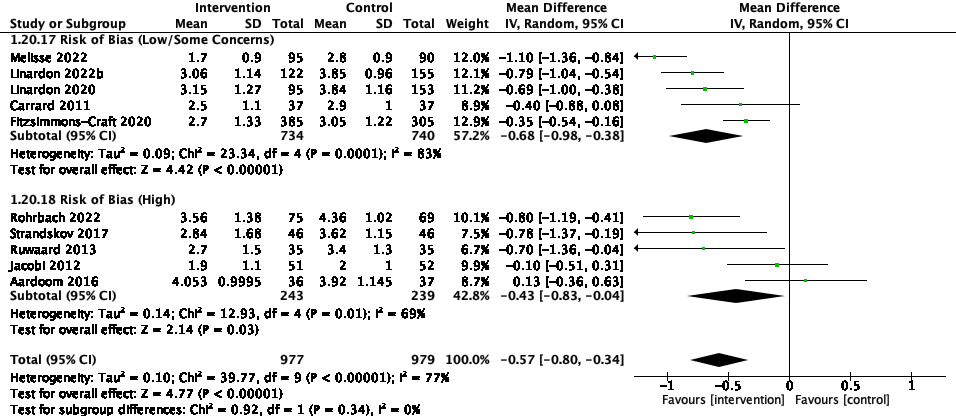
**

**Figure S6.** Funnel plot for EDE-Q Total Outcomes for WL/TAU Studies


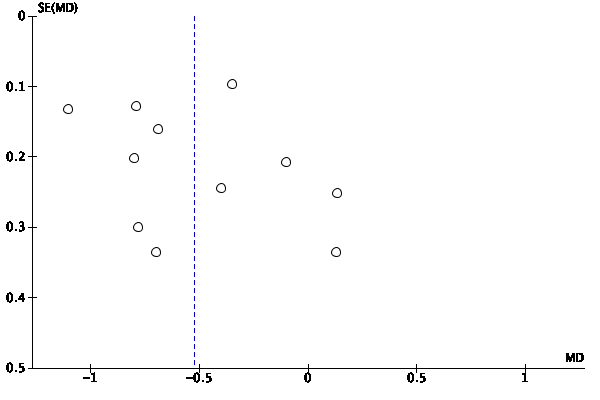

Supplement: Multimedia Appendix 2 [file mental_v11i1e57577_app2.docx]
