# Supplementary material for: Behavior Change Techniques Within Digital Interventions for the Treatment of Eating Disorders: Systematic Review and Meta-Analysis
Source: JMIR Ment Health. 2024 Aug 1;11:e57577. doi: 10.2196/57577 (PMC11327638; doi:10.2196/57577)
Supplement: Multimedia Appendix 3 [file mental_v11i1e57577_app3.docx]

**Table S1.** Application of Abbreviated Theory Coding Scheme (Michie & Prestwich, 2010) ([^43^](#_ENREF_43)) to All Studies

**Table S2**. Modes of Delivery within Included Digital ED Interventions

**Table S3.** Risk-of-Bias 2 Assessment
