# Supplementary material for: Behavior Change Techniques Within Digital Interventions for the Treatment of Eating Disorders: Systematic Review and Meta-Analysis
Source: JMIR Ment Health. 2024 Aug 1;11:e57577. doi: 10.2196/57577 (PMC11327638; doi:10.2196/57577)
Supplement: Multimedia Appendix 4 [file mental_v11i1e57577_app4.docx]

**Table S1.** Study Characteristics

| **Authors: Publication year; Country** | **Aim** | **No. of participants** | **Age in years / Ethnicity** | **ED symptoms / Baseline demographics** | **Inclusion/ Exclusion criteria** |
| --- | --- | --- | --- | --- | --- |
|  |  |  |  |  |  |
| Carrard et al (2011) **[**15] | To evaluate the efficacy of an Internet guided self-help treatment programme for BED in a community sample | N=74  (100% females)  37 randomised to each group | Mean age=36 years (S=11.4)  (No information about ethnicity) | 58% met full BED diagnostic criteria .  Only 13.5% (N =10) had a history of treatment for their eating | Inclusion:   - Met full or subthreshold diagnosis for BED (DSM-IV). (Criteria for subthreshold inclusion was at least one OBE a week for the past 3 months) - Patients on anti-depressant medication were required to have been stable on medication for at least 3 months. |
| Ruwaard et al (2013) [13] | To assess the efficacy of an online CBT of bulimic symptoms (compared to waitlist and bibliotherapy). | N=105 participants (99% females)  Online CBT=35  Waitlist=35  Bibliotherapy=35 | Mean age=32 years  (No information about ethnicity) | Participants had a varying degree of BN symptoms.  (A formal diagnosis of BN was not a requirement.) | Inclusion:   - Age above 16 years - BMI ≥ 18 - Respondents had to report recurrent binge eating, inappropriate weight-control behaviour (in the form of purging, physical exercise or dieting) and elevated concern with body shape and weight.   Exclusion criteria included:   - no heightened risk of dissociation or psychosis, - no history of suicide attempts within the past 3 years - no drug and alcohol abuse - no concurrent psychotherapy and no indication that another psychological disorder was prevailing. |
| de Zwaan et al (2017) [16] | To assess the efficacy of internet guided-self-help (GSH-I) compared to individual face-to-face CBT | N=178  148 [87.6%] female | Mean age=43.2 years (SD=12.3)  (No information about ethnicity) | Met diagnostic criteria for full or subthreshold BED (DSM-IV-TR) established via Eating Disorder Examination–Interview (EDE-I) | Inclusion:   - 18 years or older - German speaking, - BMI: between 27 and 40. |
| Strandskov et al (2017) [55] | To investigate the effects of tailored and ACT influenced internet-based CBT for patients with BN and EDNOS. | 92 adults (89 women, 3 men)  TG=46  CG=46 | Mean age=29  (No information about ethnicity) | 36 participants fulfilled the diagnostic criteria for BN (39%); and 56 for EDNOS (61%).  21 participants (77%) had a comorbid psychiatric diagnosis, with 34.8% fulfilling criteria for generalised anxiety disorder [^90^](#_ENREF_90).  Participants had an average history of EDs of 13 years.  49% had received previous psychological treatment for eating disorders. | Inclusion:   - At least 18 years old - Met criteria for BN or EDNOS   Exclusion:   - Diagnosis of AN - BMI of 17.5 or below - Concurrent psychological treatment - Acute risk of suicidality. |
| Wyssen et al (2021) [52] | To detect patients' acceptance of the BED-Online program and evaluate the eight sessions' programs efficacy across time | 63 adults with BED (87% women’ 13% men). | Mean age=37.2 years (SD= 10.4).  Nationality:  Swiss (n=56)  German (n=6)  Austrian (n=1) | 35.9% of patients suffered from a comorbid depressive or anxiety disorder, 3.1% from another comorbid mental disorder.  43% were involved in another medical, psychological, or other treatment while participating in the BED‐Online program. | Inclusion criteria:   - a primary diagnosis of BED according to DSM‐5 - aged between 18 and 70 years - provided written informed consent.   Exclusion criteria:   - current pregnancy, - the presence of another serious psychological or medical condition warranting priority treatment - the lack of sufficient German language - lack of technical skills to access the program. |
| Tregarthen (2019) [62] | To evaluate the effectiveness of a tailored, fully automated self-help version of Recovery Record, an app developed for eating disorder management. | Tailored version (n=501) or standard version of the app (n= 458). | Mean age=34.0 (SD 12.3) (SRR)  Mage=34.9 (12.5) (TRR)  Females=903/959 (94.2%)  Caucasian = 792 (82.6%) | Participants had eating disorder symptoms and did not have access to traditional treatment options. | - Ineligible if they were using RR linked with a treatment provider. - If they were receiving treatment at least weekly from a specialist ED provider. |
| Linardon (2022) [63] | To understand whether adding  interactive functionality to an Internet-based cognitive-behavioural intervention for ED symptoms is causally linked with better outcomes. | Interactive N=148  Static: N=145 | Mean age=33.64 (SD 10.35)  92.5% female  72.7% Caucasian | The sample was highly symptomatic, with 97% reporting the presence of binge eating and all but one engaging in at least one ED behaviour over the past month.  Mean score on EDE-Q subscales was 1.5 SDs above community norms.  52% reported a history of EDs; 30% reported a current ED diagnosis. | - aged 18 years or over. - had access to the Internet.   There were no other inclusion or exclusion criteria. Although the intervention was designed as a blended indicated preventative and low-intensity intervention option, people were not included/excluded based on risk or symptom level |
| Linardon (2022) [51] | To directly compare a broad vs. focused program to determine their relative efficacy, as well as against waitlist control.  (It was hypothesized that the participants randomised to either of the digital interventions would experience greater improvements in outcomes than those randomized to the waitlist, and that the focused digital intervention would not be inferior to the broad digital intervention.) | 600 met criteria for inclusion in the study:  N=202 (control)  N=199 (broad)  N=199 (focused) | “The majority were white, educated females”. No ethnic breakdown was provided. | Participants self-reported presence of recurrent binge eating, defined as one episode per very two weeks, on average, for the past three months (all > 18 years). | Inclusion:   - aged 18 years or over. - had access to the Internet and a smartphone. - self-reported the presence of recurrent objective binge eating, defined as one episode per every two weeks, on average, over the past three months. |
| Linardon et al (2021b) [60] | A two-armed, fully remote RCT was conducted to compare a blended internet- and smartphone app-based intervention against a control condition. | 403 participants  Treatment group = 202  Control group = 203 | Mean age=33.70 years (SD 10.04)  Females = 376 (93.3%)  Ethnicity:  Caucasian = 300 (74.4%)  Multiracial= 31 (7.7%)  Asian=28 (6.9%)  Other = 44 (10.9%) | The sample was highly symptomatic. Mean EDE-Q scores were more than 1^1/2^ standard deviations above community norms.  Most participants (n = 389; 96%) reported the presence of at least one objective binge-eating episode in the last month, with 362 (90%) reporting having engaged in at least one episode on average per week. | Inclusion:   - aged 18 years or over. - had access to the internet.     No further inclusion /exclusion criteria were applied - it was expected that respondents would exhibit elevated dietary restraint levels and/or binge eating due to recruitment via the psychoeducational ED platform. |
| Linardon et al., (2020) [14] | To evaluate the acceptability and efficacy of transdiagnostic, CBT-based smartphone app (‘Break Binge Eating’) for ED psychopathology through a RCT. | N=392 Intervention (n=197) or waitlist control (n=195). | Mean age=28.95 years (SD 8.17)  Female=363 (93%)  Ethnicity: White=298 (76%)  Mixed=26 (7%)  Other=68 (17%) | Diagnostic level BED (31%) and BN (42%). ( 27% as ‘Neither’).  261 (67%) currently had received no diagnosis. | Inclusion:   - 18 years or over - owned a smartphone. - self-reported the presence of at least one OBE over the past 4 weeks. |
| Melisse et al (2023) [53] | To examine the efficacy of guided self-help CBT-E compared with a delayed-treatment control condition, through an RCT with patients with BED. | 180 participants. CBT-E:  N= 89  Delayed treatment condition (after 12 weeks): N=91. | Mean age=39.4 years (SD 13.1)  Females= 163 (90.6%)  (No information about ethnicity) | 176 were diagnosed with BED and 4 were diagnosed with OSFED-BED.  ED in the past= 30 (16.7%)  Comorbid disorder:  Mood disorders= 24 (13.3%)  No= 77 (42.2%) | Inclusion:   - >/= 18 years - DSM BED or OSFED-BED diagnosis - BMI between 19.5 kg/m2 and 40 kgs/m2   Exclusion:   - EDs other than ones specified above - Acute psychosis - Clinical depression or suicidal ideation - Having received treatment in last 6 months - Pregnancy - Use of medication which might influence eating behaviour |
| Rohrbach et al (2022) [49] | To investigate the effectiveness of Featback, , a fully automated online self-help intervention (compared to WL control). | 355 participants: Featback (n=88)  WL = 90 Featback + EXP PS (N=90)  EXP PS (N=87) | Mean age=27.8 years (SD 10.8)  Female= 343 (96.7%)  Nationality:  89.9% Dutch  9% Belgian  1.1% other | Participants at least mild self-reported symptoms of an ED (52 or higher on Weight Concern Scale / Short Evaluation of EDs)  . | Inclusion:   - BMI of 18.5 or lower - At least weekly binge eating episodes or compensatory behaviours in the past 4 weeks.   Participants with severe EDs were advised to seek professional help but were permitted to participate (given they may benefit). |
| Aardoom et al (2016) [48] | To investigate the effectiveness of an internet-based intervention for individuals with ED symptoms, called "Featback". | N=354  Featback (n=87)  Waiting list control (n=90)  Featback+low intensity therapist support=88  Featback +high intensity therapist support = 89 | Mean age=24.2 (SD 7.7)  Female= 350 (98.9%)  (No information about ethnicity) | Self-reported symptoms of AN, BN or BED.  (Participants. demonstrated severe levels of ED psychopathology:  EDE-Q scores were comparable to norms for treatment-seeking patients with an ED)  Treatment history for ED = 163 (46.0%)  Duration of ED problems = 7.1 years (SD 6.6). | Inclusion:   - Age>/= 16 years - ED symptoms (scoring >./= 52 on WCS or reporting one or more of following ED symptoms reported by SEED - >/= 1 binge per week over past 4 weeks, >/= 1 compensatory behaviour per week over last 4 weeks - BMI>=18.5 |
| Fitzsimmons-Craft et al (2020) [50] | The aim was to test the hypothesis that a digital cognitive behavioural therapy (CBT)–guided self-help program, Student Bodies–Eating Disorders (SB-ED), would significantly reduce ED psychopathology in college women screening positive for an ED (excluding anorexia nervosa), compared with referral to usual care. | 690 women with EDs  N=385 (treatment group)  N=305 (control group)  512 (74.2%) undergrads. | Mean age=22.12 (SD 4.85)  100% female  Ethnicity:  White=414 (60%)  Hispanic=120 (17.4%) | Women with binge-purge EDs (both threshold and subthreshold presentations) were recruited. | Inclusion:   - Participants screened positive for a DSM-V diagnosis of ED (except anorexia nervosa) by endorsing 6 or more episodes of binge eating, vomiting, and/or laxative or diuretic use in the past 3 months.   Exclusion:   - Participants who had anorexia nervosa. |
| Jacobi et al (2012) [54] | To evaluate the effects of the internet-based Student-Bodies+ for women with symptoms of disordered eating and/or subthreshold ED syndromes. | 126 women  N= 64 (TG)  N= 62 [^91^](#_ENREF_91) | Mean age=22.3 years (SD=2.9)  (No information about ethnicity) | At baseline, 17 (26.6%) of the participants in the intervention group and 12 (19.4%) of the participants in the control group fulfilled criteria for any subthreshold ED as defined above.  6.3% (N= 8) had received past ED treatment (4 for AN, 4 for BN)  21.4% (N=27) fulfilled criteria for a lifetime diagnosis of an ED (17 AN, 8 BN, 2 BED). | Excluded:  - no symptoms of disordered eating  - current major depression  - substance misuse/mediation  - Full syndrome ED. |
| Hogdahl (2023) [61] | This study aimed to evaluate the effects of two types of internet-based cognitive behavioural therapy and a structured day patient program (standard treatment at an eating disorder clinic). | N=120  N= 98 (internet treatment)  N= 22 (DPP) | Mean age=27.3 (7.28) ICBT)  Mage - 26.5 (6.35) (DPP)  (No information about ethnicity) | EDE-Q and CIA scores were high, and SASB self-image scores low, indicating that participants had a high ED-symptom load, impaired functioning, and a negative self-image.  Duration of ED:  11.7 years (SD 8.55) (ICPT)  9.9 (SD 7.17) (DPP).  Any comorbidity was found in 64%: ICBT 62 % and DPP 73%, | Inclusion:   - Age >/= 18 years - BMI >/= 17.5 and </= 34.   Exclusion:   - ongoing ED treatment - severe symptoms of depression or anxiety - suicide plans/attempts in past year - previous experience of ICBT or DPP. |
| Wagner (2013) [64] | To evaluate the long-term effectiveness of internet-based guided self-help (INT-GSH) compared with conventional guided bibliotherapy (BIB-GSH) in females with bulimia nervosa. | 155 participants – 126 started treatment  N=70 (TG)  N=56 [^91^](#_ENREF_91) | 100% female  Mean age=24.2 (4.5) (TG)  Mean age=25.0 (3.8) CG (BIB-GSH)  (No information about ethnicity) | 90% had BN (severe)  10% had EDNOS  1/3 had a diagnosis of previous AN (before BN)  Long eating disorder histories (mean>8 years)  2/3 had engaged in previous psychotherapy.  (Note: Participants were recruited via the ED unit and had more severe BN than in some of the other studies at baseline:  Mean OBE (INT-GSH)= 32.49  Mean OBE [^92^](#_ENREF_92) = 33.42. | Inclusion:   - age 16–35 years - fulfilment of the diagnostic criteria for bulimia nervosa purging type according to DSM-IV-TR,22 EDNOS with binge eating or purging behaviour between once and twice a week or for less than 3 months - body mass index (BMI) above 18.   Exclusion:   - acute suicidality - severe depression or other mental disorders affecting cognition. - current drug misuse - current participation in CBT. |
