# Supplementary material for: Behavior Change Techniques Within Digital Interventions for the Treatment of Eating Disorders: Systematic Review and Meta-Analysis
Source: JMIR Ment Health. 2024 Aug 1;11:e57577. doi: 10.2196/57577 (PMC11327638; doi:10.2196/57577)
Supplement: Multimedia Appendix 5 [file mental_v11i1e57577_app5.docx]

**Table S1.** Summary of Interventions including Behaviour Change Techniques and Modes of Delivery Used

| **Authors: Year** | **Intervention (Condition)** | **Description of intervention** | **Behaviour Change Technique (BCTs)** | **Modes of Delivery** | **Control group** | **Duration of therapy**  **(incl follow-up)** | **Key Outcomes**  **measured** |
| --- | --- | --- | --- | --- | --- | --- | --- |
|  |  |  |  |  |  |  |  |
| Carrard et al (2011) [15] | SALUT BED (Internet-delivered CBT programme)  (BED) | 11 modules inspired by CBT techniques for binge eating treatment.  Self-observation was a key feature: a self-monitoring diary was introduced from the second module and used for the duration of the self-help treatment. | 1.2 Problem solving  1.4 Action planning  2.2 Feedback on behaviour  2.3 Self-monitoring of behaviour  2.4 Self-monitoring of outcomes of behaviour  3.1 Social support (unspecified)  3.2 Social support (practical)  4.2 Information about antecedents  5.4 Monitoring of emotional consequences  7.7 Exposure  8.1 Behavioural practice / rehearsal  9.2 Pros and cons  9.3 Comparison of future outcomes  13.2 Framing/reframing  15.4 Self-talk. | - Website - Email - Phone - At-a-distance (human interaction) | Waitlist control | 6 months  (12-months) | EDI-2 (incl. bulimia subscale)  EDE-Q (global and subscales)  OBEs |
| Ruwaard et al (2013) [13] | Online CBT (for BN)  (BN) | 20-week standardised programme based on existing CBT manuals for BN. Included awareness training, monitoring, planning and structuring meals, exposure and response prevention, cognitive restructuring, behaviours experiments, mirror exposure, positive self-verbalisation and relapse prevention. | 1.2 Problem solving  1.4 Action planning  2.2 Feedback on behaviour  2.3 Self-monitoring of behaviour  4.2 Information about antecedents  4.4 Behavioural experiments  7.7 Exposure  13.2 Framing/reframing   - 1. Self-talk | - Website - At-a-distance (human interaction) | Waitlist control  (Bibliotherapy*) | 20 weeks  (1-year) | EDE-Q global  OBEs (monthly)  Purging (monthly)  Body Attitude Test (BAT) |
| de Zwaan et al (2017) [16] | INTERBED (stemmed from SALUT; adapted for BED and translated into German)  BED | 11 sequential modules (~50 mins) delivered within 4 months. Each module combined psychoeducation and behavioural components. A self-monitoring diary was introduced from second module. Some modules (mindfulness and relaxation techniques) included audio files. | 1.2 Problem solving  2.2 Feedback on behaviour  2.3 Self-monitoring of behaviour  3.1 Social support (unspecified)  3.2 Social support (practical)  4.2 Information about antecedents  5.4 Monitoring of emotional consequences  7.7 Exposure  8.1 Behavioural practice / rehearsal  9.2 Pros and cons  9.3 Comparison of future outcomes | - Website - Email - Messaging | Active comparator:  Individual CBT: 20 x F2F sessions | 4 months  (6 months,  18 months after EOT) | EDE-Q (global and subscales  OBEs (monthly) |
| Strandskov et al (2017) [55] | "Internet-based CBT and ACT" | ACT-influenced internet-based CBT intervention, comprising of 8 modules. 4 ‘core’ modules were based on transdiagnostic theory using ACT-techniques. 4 ‘tailored’ modules addressed participants’ specific difficulties.  The final module summarised treatment gains, strategies to maintain these and relapse prevention. | 1.1.Goal setting (behaviour)  1.2 Problem solving  1.3 Goal setting (outcome)  1.9 Commitment  2.2 Feedback on behaviour  2.3 Self-monitoring of behaviour  3.2 Social support (practical)  3.3 Social support (emotional)  4.1 Instructions on how to perform the behaviour.  4.2 Information about antecedents  7.1 Prompts / cues  7.7 Exposure  8.2 Behaviour substitution  8.3 Habit formation  8.4 Habit reversal  11.2 Reduce negative emotions  13.4 Valued self-identity  15.3 Focus on past success | - Website - Email - Messaging - Phone - Video - Audio - Textual - At-a-distance (human interaction) | Waitlist control | 8 weeks  (no f/u) | EDE-Q (total and subscales)  PHQ-9  GAD-7  QOLI |
| Wyssen (2021) [52] | BED-Online  BED | Internet based GSH which included 11 Internet modules. 8-session guided cognitive-behavioural internet-based program and 3 booster sessions. | 1.1.Goal setting (behaviour)  1.2 Problem solving  1.3 Goal setting (outcome)  1.4 Action Planning  2.2 Feedback on behaviour  2.3 Self-monitoring of behaviour  2.4 Self-monitoring of outcomes of behaviour  3.2 Social support (practical)  4.1 Instructions on how to perform the behaviour  4.2 Information about antecedents  5.1 Information about health consequences  5.6 Information about emotional consequences | - Website - Email - Messaging - Textual - At-a-distance (human interaction) | WL control (NB started active treatment at 4 weeks) | 8 weeks  (6 months, 1.5 years) | EDE-Q global  OBEs (weekly / item 5 EDE-Q) |
| Tregarthen (2019) [62] | Recovery Record  (Any EDs) | “Recovery record” app:  (a) tailored version of app or (b) standard version of app. | 1. Tailored:   1.1 Goal setting (behaviour)  1.2 Problem solving  1.3 Goal setting (outcome)  1.4 Action planning  1.5 Review behavioural goal  1.7 Review outcome (goal)  2.3 Self-monitoring of behaviour  2.4 Self-monitoring of outcomes of behaviour  4.2 Information about antecedents  7.1 Prompts / cues  8.1 Behavioural practice / rehearsal  8.4 Habit reversal  9.2 Pros and cons  9.3 Comparison of future outcomes  13.2 Framing / reframing  15.3 Focus on past success.  (b) Standard:  1.1 Goal setting (behaviour)  1.2 Problem solving  1.3 Goal setting (outcome)  1.4 Action planning  1.5 Review behavioural goal  1.7 Review outcome (goal)  2.3 Self-monitoring of behaviour  2.4 Self-monitoring of outcomes of behaviour  4.2 Information about antecedents  7.1 Prompts / cues  8.1 Behavioural practice / rehearsal  13.2 Framing / reframing | - Mobile - Messaging | Active comparator: Standard version of the app | 4 weeks  8 weeks  (no f/u) | OBEs (EDE-Q Item 5) |
| Linardon (2022a) [63] | Break Binge Eating  BED/ED symptoms | Interactive version of intervention included diverse multimedia content delivery channels/’modes of delivery’ (video tutorials, graphics, written text). It also included a smartphone app component, allowing users to complete the homework exercises digitally and use progress monitoring features.  The static  version delivered identical content but via written text containing no interactive features (or app component) | 1.2 Problem solving  1.4 Action planning  2.3 Self-monitoring of behaviour  2.4 Self-monitoring of outcomes of behaviour  7.7 Exposure  5.1 Information about health consequences  5.6 Information about emotional consequences  8.1 Behavioural practice / rehearsal  8.3 Habit formation  13.2 Framing / reframing  4.1 Instructions on how to perform the behaviour  4.2 Information about antecedents | - Website - Mobile - Email - Video - Audio - Visual - Printed | Active comparator  Static version of intervention (no WL control) | 4 weeks  (no f/u) | EDE-Q (total and subscales)  OBEs (monthly) |
| Linardon (2022b) [51] | Break Binge Eating/ [Break the Diet Cycle*]  BED/ED symptoms | Delivered through smartphone app. Content based on Fairburn's transdiagnostic CBT protocol.  Included 4 modules:  the first was psychoeducational, the remaining three targeting one maintaining mechanism (dietary restraint, mood dysregulation, body image concerns).  Being self-guided, participants could decide their rate of progression.  Breaking the Diet Cycle (focused): Content was divided into four sessions, each teaching one key strategy to modify dietary restraint. Session 1) was psychoeducational in while sessions 2) taught real-time self-monitoring 3) adopting regular eating and 4) overcoming food anxiety. Delivered online and through an app, which included a digital food dairy to allow participants to monitor their eating behaviours in real-time and practice their skills. | Broad:  1.2 Problem solving  2.2 Feedback on behaviour  2.3 Self-monitoring of behaviour  2.4 Self-monitoring of outcomes of behaviour  4.1 Instructions on how to perform the behaviour  4.2 Information about antecedents  7.1 Prompts/cues  8.1 Behavioural practice/rehearsal  8.2 Behaviour substitution  11.2 Reduce negative emotions  12.4 Distraction  13.2 Framing / reframing.  Focused:  1.2 Problem solving  1.4 Action planning  2.3 Self-monitoring of behaviour  2.4 Self-monitoring of outcomes of behaviour  4.1 Instructions on how to perform the behaviour  4.2 Information about antecedents  5.1 Information about health consequences  5.6 Information about emotional consequences  7.1 Prompts/cues  7.7 Exposure  8.1 behavioural practice/rehearsal  9.3 Comparison of future outcomes  13.2 Framing / reframing | - Website - Mobile - Visual - Audio - Textual | Waitlist control | 4 weeks  (8 weeks) | EDE-Q (total and subscales)  OBEs (monthly)  Compensatory Behaviours  PHQ-4 |
| Linardon et al (2021) [60] | Break the Diet Cycle  BED/BN | Blended internet-and smartphone app intervention (see description above for focused intervention) | 1.2 Problem solving  1.4 Action planning  2.2 Feedback on behaviour  2.3 Self-monitoring of behaviour  2.4 Self-monitoring of outcomes of behaviour  2.7 Feedback on outcomes of behaviour  5.1 Information about health consequences  5.6 Information about emotional consequences  7.1 Prompts / cues  7.7 Exposure  9.3 Comparison of future outcomes  13.2 Framing / reframing | - Website - Mobile - Email - Video - Audio - Visual - Textual | Informational control group (authors website) | 4 weeks  (8 week f/u) | EDE-Q (total and subscales -not DR)  OBEs (monthly) |
| Linardon et al., (2020) [14] | Break Binge Eating (BBE)  (BED) | CBT-based smartphone app for ED psychopathology.  The BBE app comprised 4 modules, which took 30-90 mins to complete, with up to 10 short audio-recordings, reading material and various short activities.  The first module was psychoeducational, the remaining modules targeted core symptoms.  Digital self-monitoring diary allowed for monitoring of daily eating patterns and provided automated feedback on binge eating frequency and fluctuations in other key symptoms over the preceding 10 days. | 1.2 Problem solving  1.4 Action planning  2.2 Feedback on behaviour  2.3 Self-monitoring of behaviour  2.4 Self-monitoring of outcomes of behaviour  4.1 Instructions on how to perform the behaviour  4.2 Information about antecedents  7.1 Prompts / cues  8.1 Behavioural practice / rehearsal  8.2 Behaviour substitution  8.4 Habit reversal  11.2 Reduce negative emotions  12.4 Distraction  13.2 framing/reframing | - Mobile - Email - Audio - Visual - Textual | Waitlist control | 4 weeks  (8 weeks) | EDE-Q (global)  OBEs (monthly) |
| Melisse et al (2023) [53] | GSH CBT-E  BED | Guided self-help CBT-E intervention (based on "Overcoming Binge Eating: Part 2")  WL patients required to read psychoeducational section of "Overcoming Binge Eating".  The intervention included:  - psychoeducation  - daily assignments  - 2 evaluations each week. When patients did not complete their daily assignments, they received reminders. | 1.2 Problem solving  1.4 Action planning  2.2 Feedback on behaviour  2.3 Self-monitoring of behaviour  2.4 Self-monitoring of outcomes of behaviour  2.7 Feedback on outcomes of behaviour  4.2 Information about antecedents  5.1 Information about health consequences  7.1 Prompts / cues  8.2 Behaviour substitution | - Website - Phone | Waitlist control/  (Delayed treatment control - provided after 12-weeks) | 12 weeks  (24 weeks / 36 weeks) | OBEs (monthly)  EDE-Q (global, subscales) |
| Rohrbach et al (2022) [49] | (i) Featback  [(ii) Featback plus expert-patient support  (iii) Expert-patient support*]  (iv) Waiting control | Featback was a brief online self-help program that included an automated monitoring and personalised feedback system. The goal was to reduce ED symptoms by making users aware of their ED symptoms, providing support and stimulating help-seeking behaviours either professionally or in their direct environment.  It contained a summary of the problems, changes compared to previous week and guidance on how to counter the ED symptoms. Additionally users could access the Featback website for psychoeducational materials. | 1.2 Problem solving  2.2 Feedback on behaviour  2.3 Self-monitoring of behaviour  2.4 Self-monitoring of outcomes of behaviour  2.7 Feedback on outcomes of behaviour  3.1 Social support (unspecified)  3.2 Social support (practical)  3.3 Social support (emotional)  4.1 instruction on how to perform the behaviour  4.2 Info about antecedents  5.1 Info about health consequences  5.3 Information about social and environmental consequences  5.6 Info about emotional consequences  7.1 Prompts/cues  10.4 Social reward  15.1 Verbal persuasion about capability | - Website - Email - Textual | WL control / TAU | 8 weeks  (3-month /  6-month /  9-month /  12-month from post-intervention) | EDE-Q global  OBEs (monthly) |
| Aardoom et al (2016) [48] | (i) Featback  (ii) Featback+  low-intensity therapist  support  (iii) Featback+  high-intensity therapist  support  (iv) Waiting list control | (see Rohrbach, 2022) | 1.2 Problem solving  2.2 Feedback on behaviour  2.3 Self-monitoring of behaviour  2.4 Self-monitoring of outcomes of behaviour  2.7 Feedback on outcomes of behaviour  3.2 Social support (practical)  3.3 Social support (emotional)  4.1 instruction on how to perform the behaviour  4.2 Info about antecedents  5.1 Info about health consequences  5.3 Information about social and environmental consequences  5.6 Info about emotional consequences  7.1 Prompts/cues  10.4 Social reward  15.1 Verbal persuasion about capability | - Website - Email - Textual | Waitlist control/TAU | 8 weeks  (3-month /  6-month /  9-month /  12-month from post-intervention) | EDE-Q global  OBEs (monthly) |
| Fitzsimmons-Craft et al (2020) [50] | "Student Bodies - Eating Disorders", a digital CBT-guided self-help program. | Covers core components of CBT, including reducing ED behaviours (via self-monitoring and regular eating), improving body image, regulating emotions, addressing shape checking and avoidance, challenging negative thoughts and preventing relapse Program includes psychoeducational content, as well as meal planning and tracking tools, self-monitoring logs, and other interactive tools. | 1.2 Problem solving  1.4 Action planning  2.2 Feedback on behaviour  2.3 Self-monitoring of behaviour  2.4 Self-monitoring of outcomes of behaviour  2.7 Feedback on outcomes of behaviour  3.1 Social support (unspecified)  4.1 Instructions on how to perform the behaviour  4.2 Information about antecedents  5.6 Information about emotional consequences  7.7 Exposure  8.1 Behavioural practice / rehearsal  8.3 Habit formation  8.4 Habit reversal  11.2 Reduce negative emotions  12.4 Distraction  13.2 Framing / reframing | - Website - Mobile - Messaging - Textual | Referral to Usual Care / Treatment as Usual | 8-months  1-year (f/u)  2-year f/u) | EDE-Q (total)  Abstinence from all ED behaviours |
| Jacobi et al (2012) [54] | Student Bodies+ | "Student Bodies +" is an enhanced version (of Student Bodies – see above) also aimed at people with subthreshold EDs. It includes additional content related to coping with negative emotions adding skills training from dialectic behaviour therapy. The psycho-educational content on social skills and cognitive restructuring was also expanded. | 2.2 Feedback on behaviour  2.3 Self-monitoring of behaviour  2.4 Self-monitoring of outcomes of behaviour  2.7 Feedback on outcomes of behaviour  3.1 Social support (unspecified)  5.1 Information about health consequences  5.3 Information about social and environmental consequences  7.7 Exposure  8.1 Behavioural practice / rehearsal  8.2 Behaviour substitution  11.2 Reduce negative emotions  13.2 Framing / reframing | - Website - Messaging | Waitlist control | 2 months (post)  6-month follow- up | EDE-Q (total and subscales)  OBEs (monthly)  Purging  Restrictive eating |
| Hogdahl (2023) [61] | SALUT-BN  (BIB-ICBT*) | SALUT BN  There were two types of internet intervention. (SALUT-BN and BIB-ICBT). They were similar in many respects. | SALUT-BN:  1.2 Problem solving  1.4 Action planning  2.3 Self-monitoring of behaviour  2.4 Self-monitoring of outcomes of behaviour  3.1 Social support (unspecified)  4.2 Information about antecedents  5.1 Information about health consequences  8.1 Behavioural practice / rehearsal  8.2 Behaviour substitution  13.2 Framing / reframing  Day Programme (DPP):  1.1 Goal setting (behaviour)  1.2 Problem solving  1.3 Goal setting (outcome)  1.8 Behavioural contract  2.3 Self-monitoring of behaviour  2.4 Self-monitoring of outcomes of behaviour  3.2 Social support (practical)  3.3 Social support (emotional)  4.2 Information about antecedents  5.1 Information about health consequences  8.4 Habit reversal  11.2 Reduce negative emotions | - Website - Face-to-face - At-a-distance (human interaction) | Active comparator: DPP, an intensive day patient programme. | 16 weeks (tbc)  1-year (f/u) | EDE-Q total  Binge eating episodes |
| Wagner (2013) [64] | SALUT-BN | SALUT BN | 1.2 Problem solving  1.4 Action planning  2.2 Feedback on behaviour  2.3 Self-monitoring of behaviour  2.4 Self-monitoring of outcomes of behaviour  2.7 Feedback on outcomes of behaviour  3.1 Social support (unspecified)  3.2 Social support (practical)  4.2 Information about antecedents  7.7 Exposure  8.1 Behavioural practice / rehearsal  11.2 Reduce negative emotions | - Website - Email | Active comparatorBibliotherapy (BIB-GSH) | 4-months  7-months  18 months (f/u) | OBEs (monthly)  EDI-2 total score |
