# Supplementary material for: Behavior Change Techniques Within Digital Interventions for the Treatment of Eating Disorders: Systematic Review and Meta-Analysis
Source: JMIR Ment Health. 2024 Aug 1;11:e57577. doi: 10.2196/57577 (PMC11327638; doi:10.2196/57577)
Supplement: Multimedia Appendix 6 [file mental_v11i1e57577_app6.docx]

| **BCTs** | **Definition (Behaviour Change Technique Taxonomy V1)** | **Example(s) of how this was operationalised in an intervention** |
| --- | --- | --- |
| **Goals and planning** | | |
| **Action Planning** | Prompt detailed planning of performance of the behaviour (must include at least one of context, frequency, duration and intensity). Context may be environmental (physical or social) or internal (physical, emotional or cognitive) (includes | - Setting up a realistic meal schedule; Planning and structuring meals - Participants select obstacles that are relevant to them and identify actions they can take to overcome them |
| **Problem solving** | Analyse, or prompt the person to analyse, factors influencing the behaviour and generate or select strategies that including overcoming barriers and/or increasing facilitators | - Problem solving in six steps; Practicing the six steps to effective problem solving - Relapse prevention strategies; Tips for preventing relapse |
| **Feedback on behaviour** | Monitor and provide informative or evaluative feedback on performance of the behaviour (e.g. form, frequency, duration, intensity) | - A bar chart was presented so that participants could visualise their progress over the preceding 10 days - Automatic feedback, generated by the programme, provided an objective view of participants behaviour |
| **Self-monitoring of behaviour** | Establish a method for the person to monitor and record their behaviour(s) as part of a behaviour change strategy | - Digital (daily) self-monitoring food diary - Self-monitoring of meals is introduced to help the user keep track of eating patterns. |
| **Self-monitoring of outcome(s) of behaviour** | Establish a method for the person to monitor and record the outcome(s) of their behaviour as part of a behaviour change strategy | - Users monitored their weight (weekly) - "Did you binge eat?” and “Do you have an urge to binge eat?” are included in meal logs; Participants record their binge-eating frequencies each day |
| **Social Support** | | |
| **Social support (unspecified)** | Advise on, arrange or provide social support (e.g. from friends, relatives, colleagues,’ buddies’ or staff) or non-contingent praise or reward for | - The role of coaches was to provide support and answer questions. Coaches could monitor participants' progress and review exercises. |
| **Social support (practical)** | Advise on, arrange, or provide practical help (e.g. from friends, relatives, colleagues, ‘buddies’ or staff) for performance of the behaviour | - Giving instructions on how to handle unexpected emergencies (e.g., suicidality) |
| **Shaping Knowledge** | | |
| **Information about health consequences** | Provide information (e.g. written, verbal, visual) about health consequences of not performing the target behaviour | - Teaching people about the cognitive, behavioural, and affective characteristics of extreme dietary restraint, biopsychosocial consequences of extreme diets |
| **Associations** | | |
| **Prompts/cues** | 7.1 Prompts/cues Introduce or define environmental or social stimulus with the purpose of prompting or cueing the behaviour. The prompt or cue would normally occur at the time or place of performance | - Users were prompted on a daily basis during the week, at a time they selected, to review their progress - End of day prompt asking participants to record binge eating episodes |
| **Exposure** | Provide systematic confrontation with a feared stimulus to reduce the response to a later encounter | - Forbidden food exposure - Body image exercises (e.g. mirror confrontation exercises) |
| **Repetition and substitution** | | |
| **Behavioural practice/rehearsal** | Prompt practice or rehearsal of the performance of the behaviour one or more times in the context or a time when the performance may not be necessary, in order to increase habit and skill | - Users are given made up scenarios to practice - Encourage task practice of specific activities each day |
| **Self-belief** |  |  |
| **Framing / reframing** | Suggest the deliberate adoption of a perspective or a new perspective on behaviour (e.g. its purpose) in order to change cognitions or emotions about performing the behaviour | - Content around "cognitive restructuring was expanded" - Correct cognitive distortions related to eating, food, and dieting |
